# Proteograph™-based proteome and sphingolipidome analyses identified novel serum biomarkers to monitor astronauts’ health in spaceflight

**DOI:** 10.3389/fphys.2026.1773221

**Published:** 2026-04-22

**Authors:** Manuela Moriggi, Daniele Capitanio, Enrica Torretta, Ines Metatla, Petra Frings-Meuthen, Viktor Heinz, Gabor Trautmann, Michele Salanova, Dieter Blottner, Cecilia Gelfi

**Affiliations:** 1 Department of Biomedical Sciences for Health, University of Milan, Milan, Italy; 2 Laboratory of Proteomics and Lipidomics, IRCCS Ospedale Galeazzi-Sant’Ambrogio, Milan, Italy; 3 Proteomics Platform Necker, Université Paris Cité-Structure Fédérative de Recherche Necker, INSERM US24/CNRS UAR3633, Paris, France; 4 German Aerospace Center (DLR), Institute of Aerospace Medicine, Cologne, Germany; 5 Institute of Integrative Neuroanatomy, Charité - Universitätsmedizin Berlin, Berlin, Germany

**Keywords:** HDL-binding protein (HDLBP), human spaceflight, osteogenesis, ProteographTM, spaceflight serum biomarkers, sphingolipidomics

## Abstract

**Introduction:**

Long duration spaceflight leads to significant muscle mass and strength loss, which current inflight countermeasures can only partially mitigate. This study aimed to identify as yet unexplored low-abundance serological biomarkers in astronaut blood samples as meaningful biological signatures for deeper insight into musculoskeletal adaptation as complementary protocol for upgraded health monitoring in future spaceflight.

**Methods:**

Serum samples from eight long-duration mission (180 days and more) astronauts were collected pre-flight (baseline), in-flight (two time points) and post-flight (two time points). Pre- and 3/5 days post-flight samples were analyzed using the Proteograph™ XT kit, enabling the detection of protein-level differences. Mass spectrometry data were acquired in Data-Independent Acquisition mode, analyzed with Perseus using paired Student’s t-tests, and then analyzed by Ingenuity Pathway Analysis to prioritize affected pathways. Targeted serum sphingolipids quantification was carried out across all time points using Multiple-Reaction Monitoring Mass Spectrometry. Immunoblotting was performed on serum and muscle extracts from a previous space flight mission, for selected proteins.

**Results:**

Among 1,718 detected proteins, 153 showed significant changes in abundance, with 11 displaying marked alterations targeting osteogenesis (spondin, osteomodulin), lipid metabolism (perlipins 1, 3 and 4) and ECM remodelling (collagen alpha-2(XI) chain, collagen triple helix repeat containing 1). Perlipin 4, collagen alpha-2(XI) chain, and collagen triple helix repeat containing 1 were assessed by immunoblotting at all time points (pre/in/post-flight). Functional pathway analysis identified 10 pathways related to muscle function and cytoskeletal organization, and one (reelin, BDNF) associated with brain function. We also found changes in enzyme levels from pre-and post-flight muscle extracts that can be associated to decreased levels of total ceramides in the first in-flight sample (IF1), followed by a rebound in subsequent samples (IF2, POST1) showing increased glucosyl ceramide levels.

**Conclusion:**

Long-duration spaceflight induces systemic and muscle-specific changes providing deeper insights into the multifaceted mechanisms of molecular pathways related to neuro-musculoskeletal adaptation to microgravity. Identification of low-abundance serum biomarkers from astronaut blood using high-resolution and precision protocols present as novel complementary tools for broader assessment of musculoskeletal health conditions in crewed future deep space missions.

## 1 Introduction

Exposure to microgravity during long-duration space missions is associated with well-documented losses of muscle and bone mass (Genah et al., 2021; Gabel et al., 2022; Murgia et al., 2022, 2024; Blottner et al., 2023), which can only be partially mitigated by currently available inflight countermeasures. Given the limitations of current countermeasures, sensitive and system-wide monitoring tools are required to characterize physiological deconditioning and its molecular signatures in real time. Serum based candidate biomarkers identification in biological fluids represents a challenge for monitoring molecular adaptations, both during flight and throughout the recovery phase.

Blood is one of the most informative biofluids for systemic biomarker discovery. It captures signals from multiple organs, including the musculoskeletal system, which makes up about 40-50% of a human’s biomass (Hatton et al., 2023). However, the serum proteome spans ~10 orders of magnitude in protein concentration, from albumin (35–50 mg/mL) to picogram-per-mL proteins (Anderson and Anderson, 2002; Nanjappa et al., 2014). This dynamic range likely obscures a set of low-abundance signal proteins and biomolecules otherwise marked by known dominant serum biomarkers and highly complicates both discovery and decryption of as yet unknown, potentially useful serum biomarkers for deeper insight in complex human body adaptation mechanisms.

The proteins identified so far in blood represent only a small fraction of the entire serum proteome. Prefractionation methods have been adopted to reduce the dynamic range of serum proteins prior to liquid chromatography coupled with mass spectrometry (LC-MS/MS) analysis (Keshishian et al., 2017). However, these approaches often involve complex sample preparation procedures, including immunodepletion of the most abundant proteins followed by chromatographic separation. The introduction of automated technologies based on functionalized nanoparticles (NPs) represents a significant advancement in the detection of low-abundance proteins within complex biological matrices, enabling more efficient recognition and identification of potential new biomarkers from serum samples (Blume et al., 2020).

NPs in the Proteograph™ platform are specifically designed and engineered to synergistically analyze complex proteomes by exploiting the native physicochemical properties of proteins and their unique nano-bio interactions (Blume et al., 2020; Huang et al., 2023; Roger et al., 2025). This methodology enables the fractionation of serum proteins by capturing quantitative differences at protein level, for example, in small blood samples also from crew before and after a long-duration space mission.

Spaceflight proteomics studies using biological fluids (urine, plasma/serum, and secretome), consistently showed that long- and short-duration missions produced robust changes in several protein pathways linked to fluid balance, inflammation, coagulation, mitochondrial function and tissue remodeling (Binder et al., 2014; Brzhozovskiy et al., 2017; Houerbi et al., 2024).

The integrated NASA Twins multi-omics work revealed increased levels of collagen alpha-1(III) chain (COL3A1) and collagen alpha-1(I) chain (COL1A1) in urine. In addition, elevated plasma levels of apoliprotein B (APOB) and apolipoprotein A1 (APOA) were observed during the last six months duration of a flight mission compared with preflight and early inflight samples (Garrett-Bakelman et al., 2019). A recent study conducted on six-months astronaut cohort, highlighted bone-related markers using depletion-based serum samples followed by LC-MS/MS and data independent acquisition (DIA). Among them, alkaline phosphatase (ALPL), COL1A1, osteopontin (OPN), and periostin (POSTN) were significantly changed underscoring extracellular matrix (ECM) and bone remodeling (Kimura et al., 2024).

Lipid signaling regulates several cellular key processes such as cell communication, tissue repair and immune response. More specifically, sphingolipids located at the cellular membrane act as bioactive molecules regulating stress response and programmed cell death. There is limited direct research on sphingolipids (e.g., ceramides, sphingomyelins) in astronauts. However, several simulated microgravity experiments in various cell and organoid model systems showed remodeling of sphingolipids under low-gravity conditions, which may link to stress, membrane changes, and possibly inflammation/apoptosis (Çelen et al., 2023; Manis et al., 2023; Fava et al., 2024; Tolle et al., 2024). Sphingolipids can be detected and quantified in serum samples by targeted LC-MS/MS (Torretta et al., 2018, 2019, 2021a, 2021b; Barbacini et al., 2019, 2022a, 2022b; Al-Daghri et al., 2020).

The primary objective of this study is the identification of novel putative biomarkers characterizing human body adaptation to spaceflight, which can also serve as complementary strategies for improved and updated monitoring of crew health in future missions. Building on this, the study aims to broaden the range of detectable serum molecules, including sphingolipids, by employing robust high-resolution protocols, essential for gaining a more comprehensive understanding of the composition of this complex biofluid. We assumed that fluctuations of low-abundance biomarkers in the small blood samples obtained from astronauts under operational constraints could reflect novel, yet unidentified biological signatures associated with extreme physiological adaptation during spaceflight.

## 2 Materials and methods

### 2.1 Sample study design and participants

Blood samples were obtained from eight astronauts (5 males, 3 females; age 47 ± 5.6 years) recruited from the National Aeronautics and Space Administration (NASA) and European Space Agency (ESA) astronaut corps. Detailed characteristics are provided in **Supplementary Table S1**. Samples were collected at five time points: one pre-flight (PRE, 60 days before launch), two in-flight (IF1, days 31–60; and IF2, 10 days before return), and two post-flight (POST1, 3–5 days after landing; and POST2, 105 days after landing). Aliquots of the blood samples used in this study originated from an ESA commissioned ISS experiment (Schoenrock et al., 2024).

### 2.2 Sample collection and storage

Blood samples were drawn by cubital venipuncture (sterile 4.5 ml BD SSTTM, ref.368879). After blood coagulation, samples were centrifuged at 3,000 rpm for 10 minutes and immediately frozen in liquid nitrogen (BDC on the ground samples) or stored deep frozen in the onboard cold storage at minus 80 °C (flight samples). The deep frozen vials were shipped to the PI´s laboratory (Charité Berlin, Germany) and cut still in frozen state with a dedicated RNase-cleaned PVC pipe cutter as operated in a minus 20 °C cooling compartment (CM 1850 Cryostat, leicabiosystems.com) in order to separate the pelleted blood cell fraction (including the rich platelet plasma) from the low-platelet serum supernatant. The still frozen serum supernatant was recovered in bloc in 15 ml sterile FalconTM tubes each (thermofisher.com), aliquoted in 1.5 ml Eppendorf cups, and stored frozen in a minus 30 °C freezer. No signs of hemolysis and lipemia were present. From the entire collection of 40 samples per each participant (n=8, 5 time points) only a small amount of material (300 µl each sample) was available for this study requiring accurate management of the experimental design.

### 2.3 Inflight exercise

A list of inflight countermeasure days and protocol for Astronauts A-I during their stay on the International Space Station (ISS) is published elsewhere (Schoenrock et al., 2024). All astronauts (n=8) performed regular inflight exercise during the 6 months stay on ISS. Inflight exercise duration and standard protocol: approx. 2.5hr/day using either of the three exercise modes (T2=endurance/CEVIS=cardiovascular/ARED=resistive muscle) (Loehr et al., 2015; Scott et al., 2020).

### 2.4 Proteomic Profiling with Proteograph™ XT Analysis

The Proteograph™ Assay is a fully automated sample preparation aiming to enrich low abundance proteins in samples. Proteograph XT kit includes a panel of engineered nanoparticles with distinct physicochemical properties, allowing the fractionation of serum proteins by capturing the quantitative differences between various biological samples, thus enabling an unaltered analysis for discovering the protein-level differences in POST1 vs. PRE subjects (Roger et al., 2025). 20 sera samples (200µL each) were prepared using the Proteograph XT kit following the manufacturer’s instructions (Seer Inc, USA). The SP100 automated liquid-handling robot was used to carry out the entire protocol, from bead incubation and digestion to peptide quantification and peptide reconstitution. Briefly, each serum sample was incubated with two different nanoparticle mixtures, resulting in two fractions per sample (NPA and NPB). After incubation and nanoparticle corona formation, the nanoparticles were separated using a magnetic rack, and unbound components were removed by washing. The proteins bound to the nanoparticles were reduced, alkylated, and digested with trypsin/LysC. The digested peptides were transferred to peptide Clean-up plate. The peptides were washed, filtered and eluted into collection plates under positive pressure (MPE). Peptide quantification was automatically performed using the Pierce Fluorometric Peptide Assay (Thermo Scientific, USA). The peptides were then dried in a SpeedVac. All peptides were resuspended in 2% ACN, 0.1% formic acid in HPLC grade water, and the peptides from NPA and NPB were pooled and injected into the LC-MS/MS. A total of 600 ng of peptides were injected on an Evosep One system coupled to a timsTOF HT (Bruker Daltonics, Germany) mass spectrometer. The Evosep One system operated with 30SPD Samples Per Day (30SPD) using a 15cm x 150µm Performance column (EV1137, Evosep) at 40 °C. Mass spectrometric data were acquired using the parallel accumulation serial fragmentation (PASEF) acquisition method in Data Independent Acquisition (DIA) mode with a 19-windows method using 33Da windows covering the mass ranges over a 400-1050m/z. The range of ion mobilities values from 0.67 to 1.29 V s/cm2(1/K0). The cycle time was set to 1.06s.

#### 2.4.1 Data analysis

The data analysis was performed using DIA-NN software (version 1.8.1) (Demichev et al., 2020). A search against the human UniProtKB/Swiss-Prot Homo sapiens database (downloaded July 03rd 2023, 20423 entries) was performed using the library-free mode. For this purpose, “FASTA digest for library free search/library generation” and “Deep learning spectra, RTs and IMs prediction” options were checked for precursor ion generation. The library-free search parameters included digestion with trypsin, tolerating the omission of one cleavage site, and allowing 2 variable modifications. Carbamidomethylation (Cys) was set as the fixed modification, whereas protein N-terminal methionine excision, methionine oxidation and N-terminal acetylation were set as variable modifications. The peptide length range was set to 7–30 amino acids, precursor charge range 2-4, precursor m/z range 300-1800, and a fragment ion range of 200–1800 m/z. The false discovery rates (FDRs) at the protein and peptide level were set to 1%. Match between runs was allowed. For the quantification strategy, Robust LC (high precision) was used as advised in the software documentation, whereas default settings were kept for the other algorithm parameters. Data are available via ProteomeXchange with identifier PXD069732.

#### 2.4.2 Statistical analysis

Proteomic data were analyzed using Perseus software (version 2.02.11) (Tyanova et al., 2016). Protein intensities were log2-transformed to stabilize variance. Proteins were retained only if they had valid values in at least 80% of samples within each group; proteins with excessive missing values were excluded. Remaining missing values were imputed by generating a Gaussian distribution with a standard deviation of 33% of the measured values’ standard deviation and a downshift of 1.8 standard deviations, to simulate low-abundance signals. Statistical comparisons between POST1 and PRE samples were performed using a paired Student’s t-test, applying a FDR threshold of 0.05 to identify significantly changing proteins.

#### 2.4.3 Ingenuity pathway analysis

Functional and network analyses of statistically significant protein expression changes were performed using IPA software (Qiagen, Hilden, Germany, Summer Release 2024). The “Core Analysis” function was employed to understand the significance of data by examining canonical pathways enriched with differentially regulated proteins. p-values were calculated using a right-tailed Fisher’s exact test, and the activation z-score was used to predict pathway activation or inhibition. Pathways with a Fisher’s exact test p-value < 0.05 and a z-score ≤ −2 or ≥ 2 were considered statistically significant.

### 2.5 Sphingolipid analysis

#### 2.5.1 Sphingolipids reagent and chemicals and extraction

##### 2.5.1.1 Reagents and chemicals

LC-MS analytical grade water and methanol were from Thermo Fisher Scientific. LC-MS grade ammonium formate, formic acid and acetic acid, as well as 3,5-Di-tert-4-butylhydroxytoluene (BHT), were from Sigma-Aldrich (Saint Louis, MO, USA). HPLC analytical grade ethanol and chloroform were from VWR. Potassium hydroxide was from Merk Millipore (Burlington, MA, USA). Sphingosine (d17:1), sphinganine-1-phosphate (d17:1), ceramide (d18:1/12:0), sphingomyelin (d18:1/12:0) and glucosyl (β)ceramide (d18:1/12:0) were from Avanti Polar Lipids (Alabaster, AL, USA).

##### 2.5.1.2 Sphingolipid extraction

Sphingolipids were extracted as previously described, with minor modifications (ref). Sphingolipids were extracted from the sera of eight astronauts at five time points: PRE, IF1, IF2, POST1 and POST2. 50 ul plasma were fortified with 200 pmol of each internal standard (sphingosine (d17:1), sphingosine-1-phosphate (d17:1), ceramide (d18:1/12:0), sphingomyelin (d18:1/12:0), and glucosyl (β)ceramide (d18:1/12:0) and mixed with 0.1 mL of LC-MS water and 1.5 mL of methanol/chloroform 2:1. Samples were briefly sonicated and heated at 48 °C overnight. Then, 0.15 mL of potassium hydroxide (KOH) 1 M in methanol was added to every sample for saponification, that was neutralized with 0.15 mL of acetic acid 1 M after 2-h incubation at 37 °C. Samples were dried, resuspended in methanol, and centrifuged for 3 min at 10,000×g. Supernatants were collected in UPLC glass vials and stored at −80 °C until analyses.

#### 2.5.2 Sphingolipids LC-MS/MS analysis

Ceramides, dihydroceramides, sphingomyelins, dihydrosphingomyelins and glucosylceramide were detected by Multiple-Reaction Monitoring Mass Spectrometry (MRM-MS) using a Xevo TQ-S micro mass spectrometer (Waters, Milford, MA, USA). 2 µl of extract were injected and separated on a C8 Acquity UPLC BEH (Waters, Milford, MA, USA), 100 mm × 2.1 mm id, 1.7 µm (Waters), kept at 30 °C, using the following linear gradient: 0.0 min: 15% B; 1 min: 30% B; 1.10 min: 70% B; 4.50 min: 70% B; 4.51 min: 99% B; 5 min: 99% B; 5.10 min: 15% B; 6.60 min: 15% B at 0.3 ml/min flow rate. Phase A was 2 mM ammonium formate in acetonitrile/water (60:40, v/v) with 0.1% formic acid while phase B consisted of 2 mM ammonium formate in isopropanol/acetonitrile/water (90:9:1, v/v/v) with 0.1% formic acid. An electrospray interface operating in positive ion mode was employed to obtain MS/MS spectra by acquiring MRM transitions (**Supplementary Table S2****).** The capillary voltage was set at 3.5 kV. The source temperature was set to 150 °C. Desolvation gas flow was set to 1000 and desolvation temperature was set to 350 °C. Data were acquired by MassLynx ™ 4.2 software and quantified by TargetLynx software. Results were normalized to protein content, determined by bicinchoninic assay (BCA Pierce).

#### 2.5.3 Statistical analysis

Differences in Cers, dhCers, SMs, dhSMs and HexCers levels were assessed among groups, with a semiquantitative approach, by comparing abundance of SLs across samples. Statistical analysis was performed using GraphPad Prism Software v.8.0.2. We assessed data normality using the Shapiro–Wilk test (α = 0.05). When normality was confirmed, we applied ANOVA (p < 0.05) followed by Tukey’s *post-hoc* test; otherwise, a non-parametric test (Friedman, p < 0.05) was performed, followed by Dunn’s multiple comparisons test. The coefficient of variation (CV) was assessed to be below 10%.

### 2.6 Immunoblotting

#### 2.6.1 Quantification of selected molecules in serum

Protein extracts (50 µg) from PRE, IF1, IF2, POST1 and POST2 serum samples were loaded and resolved on 5-12% and 5-14% gradient polyacrylamide gels. Blots were incubated with antibodies bought by the Santa Cruz Biotechnology (Dallas, TX, USA), Invitrogen and Assay Genie (Dublin, Ireland): mouse monoclonal anti-collagen triple helix repeat containing 1 (CTHRC1), 1:1000; rabbit polyclonal anti-perilipin 4 (PLIN4), 1:1000; rabbit polyclonal anti-type XI collagen with two alpha chains (COL11A2), 1:1000; mouse monoclonal anti-HDL binding protein (HDLBP), 1:500. Full-length images are available in **Supplementary Figure S1**.

#### 2.6.2 Quantification of sphingolipid enzymes in muscle extracts

Protein skeletal muscle extracts (50 μg) from one short-duration mission, SDM (acute exposure, no inflight exercise) and two long-duration mission, LDM astronauts (chronic exposure, with regular inflight exercise), as previously described (Blottner et al., 2023), were loaded in duplicate and resolved on 12–16% gradient polyacrylamide gels. Blots were incubated with primary antibodies as follows: mouse monoclonal anti-serine palmitoyltransferase 1 (SPTLC1), 1:500; mouse monoclonal anti-glucosylceramide synthase (UGCG), 1:500; mouse monoclonal anti-sphingosine kinase 2 (SPHK2), 1:500. Full-length images are available in **Supplementary Figure S1**.

#### 2.6.3 Protocol and statistics

After washing, membranes were incubated with anti-rabbit (GE Healthcare, 1:10,000) or anti-mouse (Jackson ImmunoResearch, Ely, UK, 1:5000) secondary antibody conjugated with horseradish peroxidase. Signals were visualized by chemiluminescence using the ECL Prime detection kit and the Image Quant LAS 4000 (GE Healthcare) analysis system. Band quantification was performed using ImageQuant TL v. 8.1 (GE Healthcare) and statistical analyses were conducted with GraphPad Prism v. 8.0.2. Serum samples were analyzed by one-way ANOVA followed by Tukey’s *post hoc* test (p < 0.05), whereas skeletal muscle samples were evaluated using paired t-test (p < 0.05). Band intensities were normalized to total protein content detected by Sypro Ruby staining.

## 3 Results

The analysis of serum samples aimed to identify circulating markers potentially linked to the proteomic and sphingolipidomics variations observed in previous studies of the muscle proteome and nitrosoproteome associated adaptation to prolonged microgravity in spaceflight and in bed rest subjects (Blottner et al., 2023, 2024, Barbacini et al., 2022b). To achieve this objective, we implemented a novel approach that expands the dynamic range of detectable species in the sera of subjects exposed to extended periods of microgravity. Experimental design is explained in **Supplementary Figure S2**.

### 3.1 Proteomic profiling of serum before and after spaceflight using Proteograph™

**Figure 1:**
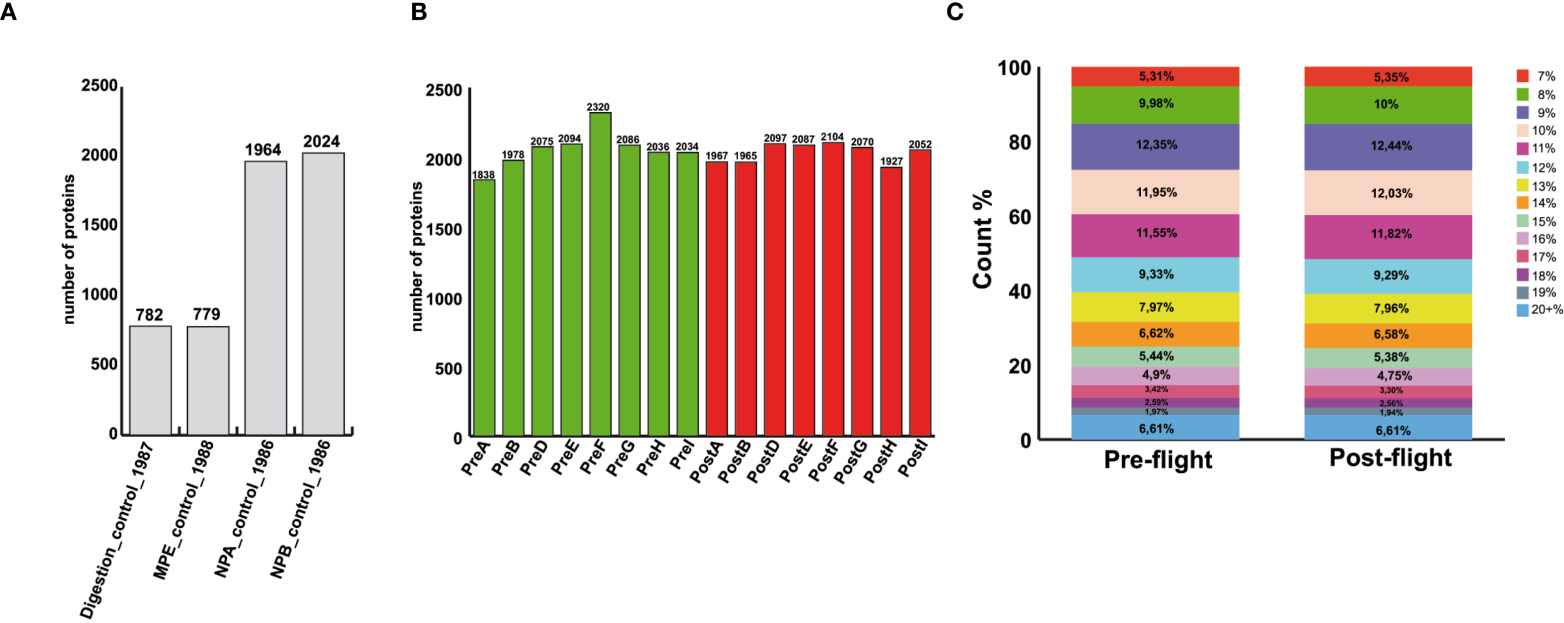
Proteograph™ quality controls. **(A)** Number of proteins identified in the four intra-experiment quality controls performed on Seer QC plasma, confirming successful trypsin digestion of neat serum (digestion_control), correct desalting (MPE_control), and effective enrichment using both nanoparticles (NPA_ and NPB_controls). **(B)** Bar chart showing the number of proteins identified in each serum sample following nanoparticle-based enrichment. **(C)** Distribution of peptide lengths in samples collected pre- and post-flight (PRE and POST1 groups), demonstrating consistent sample quality across the two collections.

In our study, PRE and POST1 serum samples from eight subjects were analyzed. The Proteograph™ analysis kit was utilized for the enrichment of low-abundance serum proteins, followed by mass spectrometry analysis. All the quality controls metrics of the Proteograph assay are reported in **Figure 1**, **Supplementary Figure S3**.

We identify a set of proteins with valid intensity values present in at least 80% of the samples within each group resulting in the identification of 1,718 proteins detected in at least seven individuals. Statistical analysis, including the fold change values for all altered proteins, together with their statistical significance (p- and q-values), revealed that 153 proteins exhibited significant changes when comparing POST1 to PRE samples (**Supplementary Table S3**). After correcting for false positives using the permutation-based FDR method, 11 proteins were significant (paired t-test, FDR = 0.05). These proteins are considered candidate biomarkers for their monitoring pre, in and post-flight (**Table 1**).

**Table 1:** A short list of eleven proteins with significant changes after permutation-based FDR correction.

Protein ids	Protein description	Genes	% fold change POST1 vs PRE	Serum concentration
O60240	Perilipin-1	PLIN1	192	22 ng/L (MS)
Q96A32	Myosin regulatory light chain 11	MYL11	187	1 µg/L (MS)
Q96Q06	Perilipin-4	PLIN4	182	55 ng/L (MS)
P29120	Neuroendocrine convertase 1	PCSK1	150	
Q99983	Osteomodulin	OMD	69	21 µg/L (MS)
O60664	Perilipin-3	PLIN3	68	11 µg/L (MS)
Q9BUD6	Spondin-2	SPON2	62	2.6 µg/L (MS)
P13942	Collagen alpha-2(XI) chain	COL11A2	32	2.1 µg/L (MS)
Q96CG8	Collagen triple helix repeat-containing protein 1	CTHRC1	20	420 ng/L (MS)
P00739	Haptoglobin-related protein	HPR	-16	49 mg/L (IA)
P02776	Platelet factor 4	PF4	-37	3 ng/L (IA)

Included are UniProt ID, protein name, gene symbol, % fold change (POST1 vs PRE), serum concentration (Human Protein Atlas), and quantification method (MS, mass spectrometry; IA, immunoassay). PRE, pre-flight; POST1, three-five days after landing.

The data indicates increased levels of perilipins-1, 3, and 4 (PLIN1, PLIN3, PLIN4) post-flight. Even low concentrations of these proteins in the bloodstream may suggest alterations in lipid metabolism and the presence of inflammation (Chandrasekaran et al., 2024). Evidence of cartilage remodeling is suggested by the increase of COL11A2, an ECM-specific protein critical for cartilage collagen fibrils formation and ECM organization. Additionally, CTHRC1, ECM-associated protein involved in vascular remodeling and bone formation, was observed. CTHRC1 can influence ECM deposition, remodeling, and interactions with other ECM components. Spondin-2 (SPON2), another ECM protein, positively regulates bone metabolism by activating WNT/β-catenin signaling (Knight et al., 2018). Circulating osteomodulin (OMD), involved in osteogenesis through BMP2/SMAD signaling, was also increased (Lin et al., 2021). Among contractile proteins, myosin regulatory light chain 11 (MYL11) stands out in this analysis; it is a regulatory subunit of myosin that plays a crucial role in maintaining muscle integrity during development. In addition to these findings, neuroendocrine convertase 1 (PCSK1) was found to be elevated in POST1 samples. This enzyme is involved in the proteolytic processing of various protein precursors, primarily neuropeptides and prohormones, and directly affects glucose metabolism, insulin sensitivity, and appetite regulation. Cleavage of these protein precursors is essential for their biological activity, including the maintenance of glucose homeostasis (Ramos-Molina et al., 2016; Verde et al., 2025).

Conversely, two proteins, haptoglobin-related protein (HPR) and platelet factor 4 (PF4), were found to be downregulated in POST1. HPR binds hemoglobin with high affinity and functions as an innate immune molecule, particularly in association with high-density lipoproteins (HDL) containing apolipoprotein L1 (Nielsen et al., 2006). PF4 is a multifunctional protein that plays a key role in regulating coagulation and inflammatory responses (Ji et al., 2025).

### 3.2 Functional and pathway analysis of serum proteins altered by spaceflight

The dataset comprising 153 proteins, including protein identifiers, statistical p-values, and fold change values, was subjected to bioinformatics enrichment analysis using Ingenuity Pathway Analysis (IPA) software (Qiagen, Hilden, Germany). IPA enables functional and network analysis of statistically significant changes in protein expression. The “core analysis” function was applied to as hypothesis-generating to avoid mechanistic over-interpretation of biological processes, canonical pathways, diseases, and functions enriched with differentially regulated proteins. Significance was determined using a right-tailed Fisher’s exact test. Additionally, the z-score, which accounts for the directionality of the observed effects, was applied to predict activation or inhibition of pathways, upstream regulators, diseases, and biological functions. A Fisher’s exact test p-value < 0.05 and a z-score ≥ 2 were considered statistically significant.

**Figure 2:**
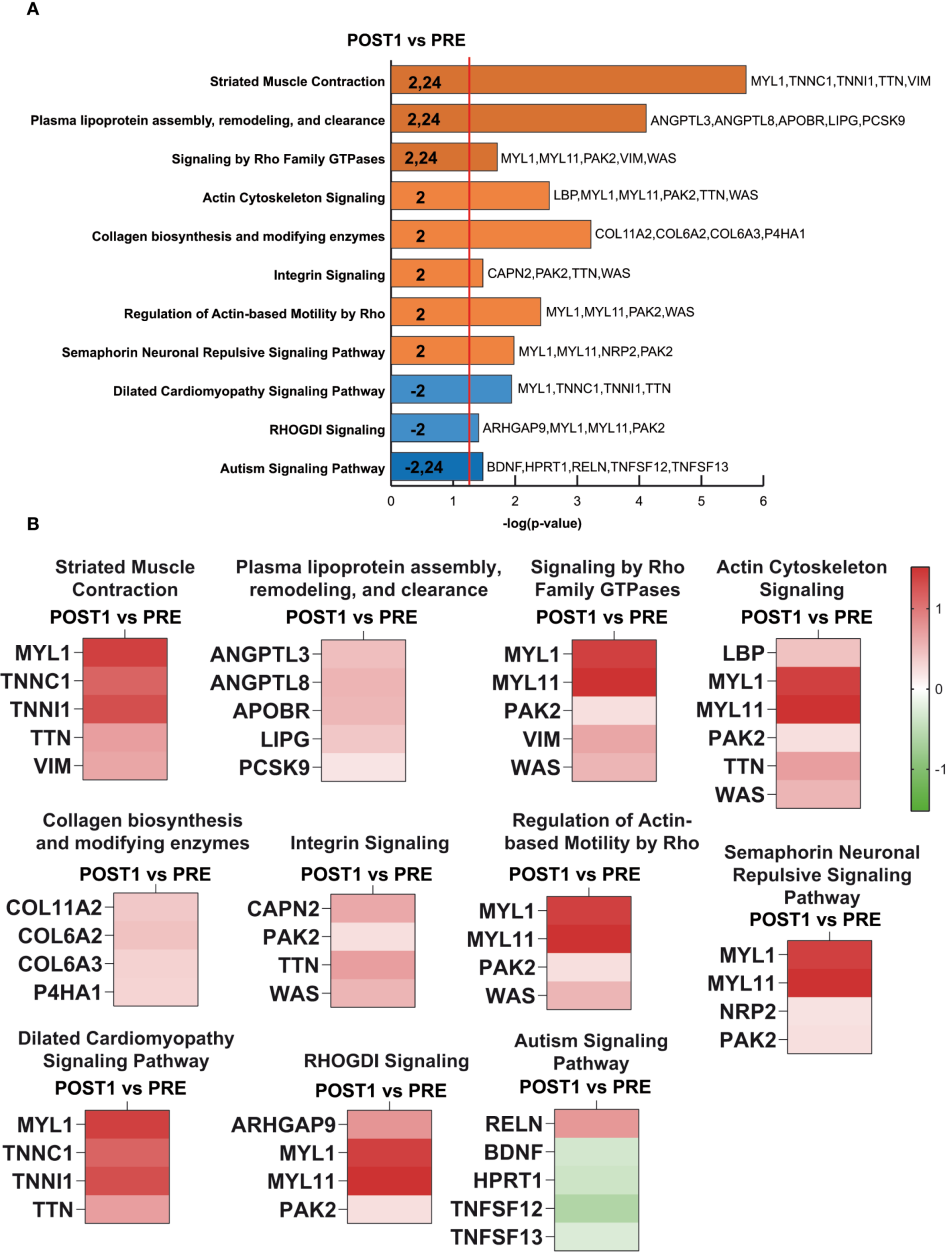
IPA, Ingenuity Pathway Analysis. **(A)** IPA of canonical pathways ranked by z-score. The orange vertical line indicates the significance threshold (p = 0.05). Orange bars represent predicted pathway activation, while blue bars indicate predicted inhibition, based on z-score values (activation: z ≥ 2; inhibition: z ≤ −2). **(B)** Heatmap showing the relative abundance of proteins grouped according to canonical pathways. Green and red indicate statistically significant decreases or increases, respectively, in protein levels (POST1 vs. PRE; paired Student’s T-test, p < 0.05) derived from the proteomics dataset.

The analysis focused on canonical pathways, revealing ten pathways associated with muscle function and cytoskeletal organization, and one related to brain function. Notably, molecules detected in the blood were associated with activation of collagen biosynthesis, actin cytoskeletal signaling, regulation of actin-based motility by Rho, semaphorin-mediated neuronal repulsive signaling, integrin signaling, striated muscle contraction, plasma lipoprotein assembly, remodeling, and clearance, as well as RhoGTPase-mediated signaling. Conversely, pathways related to muscle function, including dilated cardiomyopathy and RhoGDI signaling, were predicted to be inhibited. In terms of brain function, a reduction in signaling can be hypothesized, the glycoprotein reelin (RELN) and brain-derived neurotrophic factor (BDNF), associated with neuronal migration, proliferation, and differentiation and involved in neuroplasticity and neuromuscular adaptation was observed. These findings are summarized in **Figure 2**.

The heatmaps in **Figure 2B** illustrate the increased or decreased abundance of proteins associated with each canonical pathway. As shown, most proteins released into the serum are directly related to muscle function, many of them associated with the loss of muscle strength commonly observed in astronauts after prolonged exposure to microgravity, which is not compensated by countermeasures. These include troponin I (TNNI1), myosin light chain 1 (MYL1), vimentin (VIM), actinin alpha 4 (ACTN4), collagen type VI alpha 1 chain, alpha 2 chain and alpha 3 chain (COL6A1, COL6A2, COL6A3). Semaphorin signaling suggests structural disorganization associated with the neuromuscular junction with increased level of MYL1, myosin light chain 11(MYL11), neuropilin 2 (NRP2) and p21(RAK) activated kinase 2 (PAK2) (Monti et al., 2021). Furthermore, regarding brain function, the inhibited signaling of the pathway suggests a subset of proteins involved in cognitive processes whose expression levels are decreased, potentially associated with alterations in post-transcriptional gene regulation mediated by RNA-binding proteins (RBPs) (Zinnall et al., 2022).

### 3.3 Targeted immunoblotting

**Figure 3:**
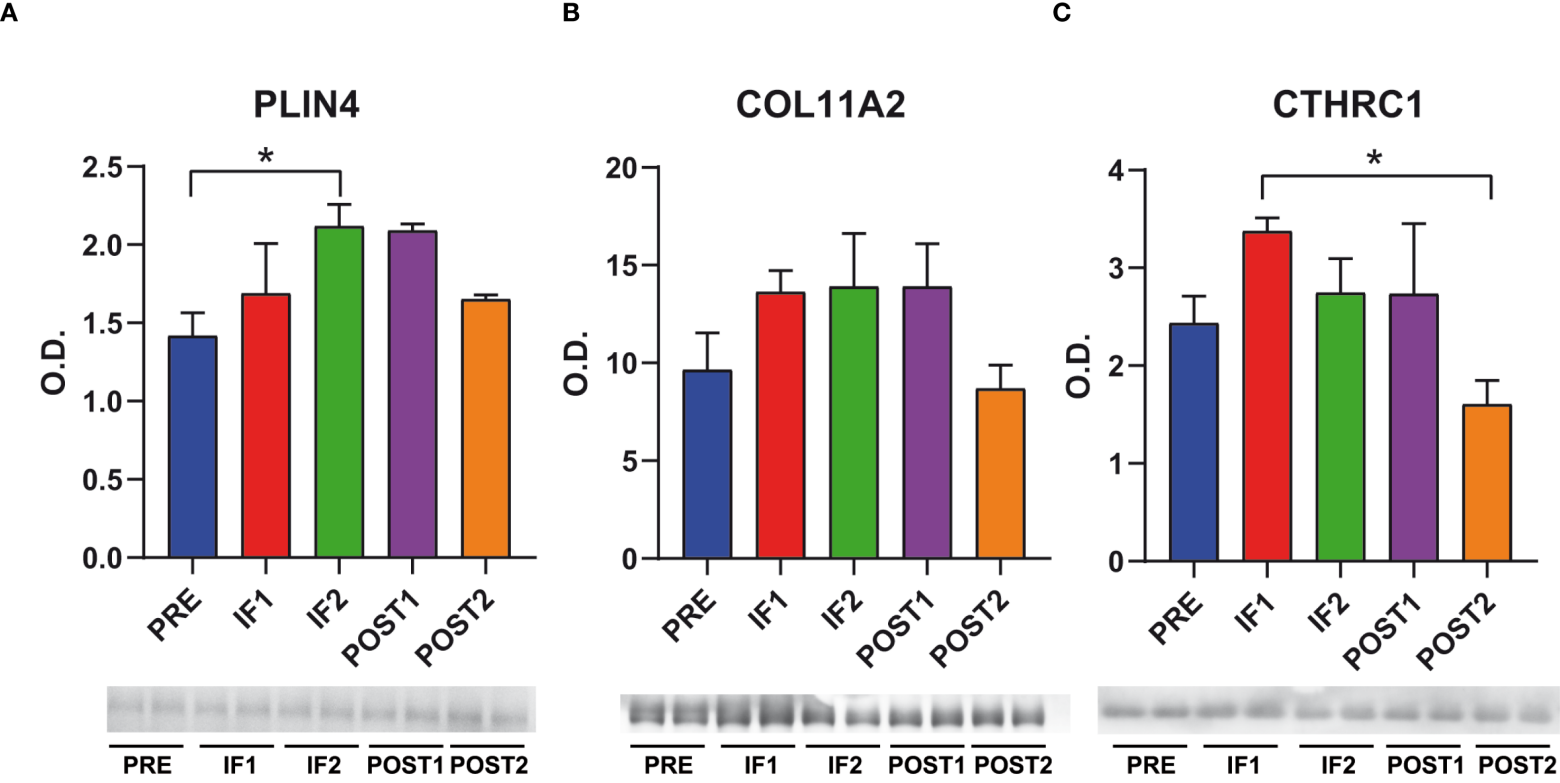
Targeted immunoblotting. Representative bar graphs (means ± SD) and corresponding immunoblot images showing quantitative changes in perilipin-4 (PLIN4), collagen alpha-2(XI) chain (COL11A2), and collagen triple helix repeat-containing protein 1 (CTHRC1) in serum across pre-flight (PRE), in-flight (IF1, IF2), and post-flight (POST1, POST2) time points. Data was normalized to the total protein load stained with Sypro Ruby. O.D. = optical density; * indicates a significant difference (*p < 0.05). Statistical analysis was performed using one-way ANOVA followed by Tukey’s *post hoc* test, with significance set at p < 0.05. Full-length images are available in **Supplementary Figure S1**.

Among the 11 candidate proteins, a subset was selected for the assessment with an independent technology and monitoring at different time points to evaluate the changes during the flight as well as the recovery after the flight. Proteins PLIN4, COL11A2, and CTHRC1 were quantified in PRE, IF1, IF2, POST1, and POST2 samples (**Figure 3**). Immunoblotting results indicated a progressive increase in PLIN4 levels from PRE to IF2, where the change was significant, levels were maintained in POST1 with a tendency to normalization in POST2. COL11A2 exhibited a similar upward trend, although the changes were not statistically significant. CTHRC1 levels increased significantly during IF1 and gradually decreased, reaching minimal values in POST2. These results reinforce the validity of the 11 candidate proteins as putative biomarkers of muscle adaptation to long term microgravity exposure.

**Figure 4:**
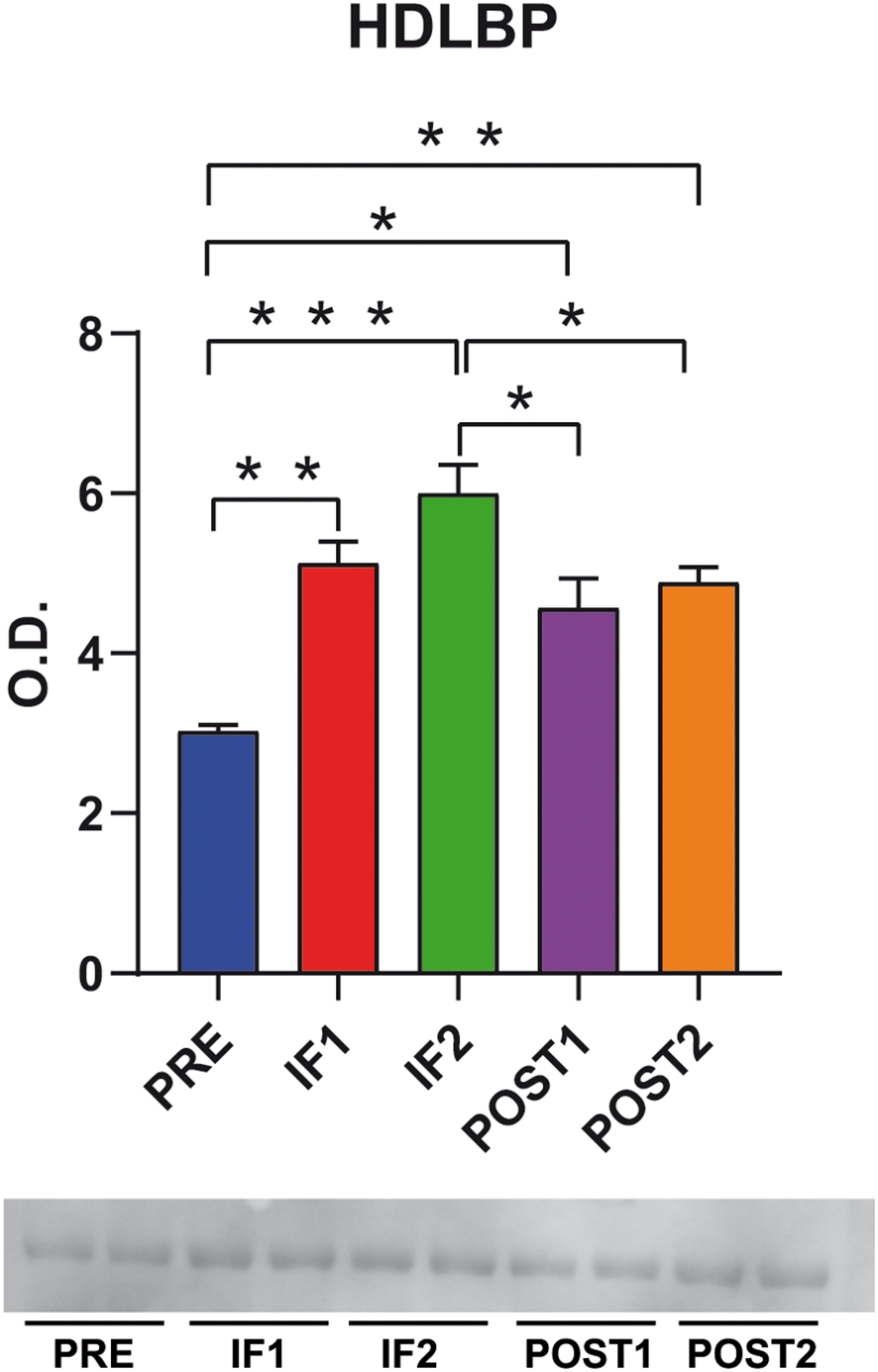
HDLBP, HDL-binding protein immunoblotting. Representative bar graph (means ± SD) and corresponding immunoblot image showing quantitative changes in HDLBP levels in serum collected pre-flight (PRE), in-flight (IF1, IF2), and post-flight (POST1, POST2). Data were normalized to the total protein load visualized with Sypro Ruby staining. O.D. = optical density; * indicates a significant difference (*p < 0.05, **p < 0.01, ***p < 0.00). Statistical analysis was performed using one-way ANOVA followed by Tukey’s *post hoc* test, with significance set at p < 0.05. Full-length blot images are provided in **Supplementary Figure S1**.

HDL binding proteins (HDLBP) plays crucial roles in heterochromatin formation, chromosome segregation, and mRNA stability, and has been associated with autism spectrum disorders (Banday et al., 2021). Notably, HDLBP functions as an RNA-binding protein with a key role in endoplasmic reticulum (ER) translation, acting as a regulatory factor in gene expression, maintaining genome stability, recovering DNA damage post irradiation, supporting mRNA transport and metabolism, and contributing to immune system development. It interacts with more than 80% of ER-localized mRNAs (Mushtaq et al., 2023). In addition, it has been associated with lipid metabolism being defined as HDL binding protein (Schaefer et al., 2014). To further investigate the potential role of HDLBP in post-transcriptional control and DNA repair, we detected its expression in our samples using immunoblotting (**Figure 4**). HDLBP levels increased in IF1 and IF2 compared to PRE, and although still elevated in POST1 and POST2, they trended toward partial normalization. These findings suggest that the upregulation of HDLBP may be associated not only with lipid metabolism but also to mechanisms of DNA repair and genome stability. Moreover, the increased abundance of HDLBP could support enhanced mRNA translation within the ER, potentially as an adaptive response to counteract ER stress.

### 3.4 Targeted sphingolipid analysis in response to spaceflight

Building on our previous studies in bedrest subjects, who underwent countermeasures with or without antioxidant treatment and indicating decreased ceramide levels, we performed targeted lipidomics to analyze sphingolipid levels in serum (Barbacini et al., 2022).

Given the persistent increase in HDLBP, we aimed to determine whether this molecule plays a protective role in regulating the mRNA translation of enzymes synthesized in the ER. Sphingolipids were selected for this analysis because the enzymes responsible for their synthesis are in the ER, and their levels can be assessed in blood using targeted mass spectrometry. These molecules are essential components of the plasma membrane and lipid rafts, contributing to cardiovascular disease (CVD) and oxidative/nitrosative stress, factors previously recognized in our muscle proteome analyses (Blottner et al., 2023, 2024). The biosynthesis of sphingolipids begins at the cytosolic layer of the ER. Ceramide is produced through the conversion of sphinganine to dihydroceramide by ceramide synthase enzymes, which are in the ER. From the ER, ceramide can be transported to the Golgi apparatus, leading to the production of glycosphingolipids and gangliosides. Glycosphingolipids are formed by the sequential addition of one or more monosaccharides; for example, the addition of glucose produces glucosylceramide. These glycosphingolipids are then transported to the plasma membrane and incorporated into lipid rafts.

Before analyzing the spingolipidome in serum we took advantage from hints provided from a previous proteomic analysis of muscle tissue from astronauts involved in a long and short duration exposure to microgravity (Blottner et al., 2023). As exploratory analysis we evaluated changes in key enzymes involved in sphingolipid synthesis in the muscle tissue previously collected to support the analysis of the sphingolipidome in serum.

**Figure 5:**
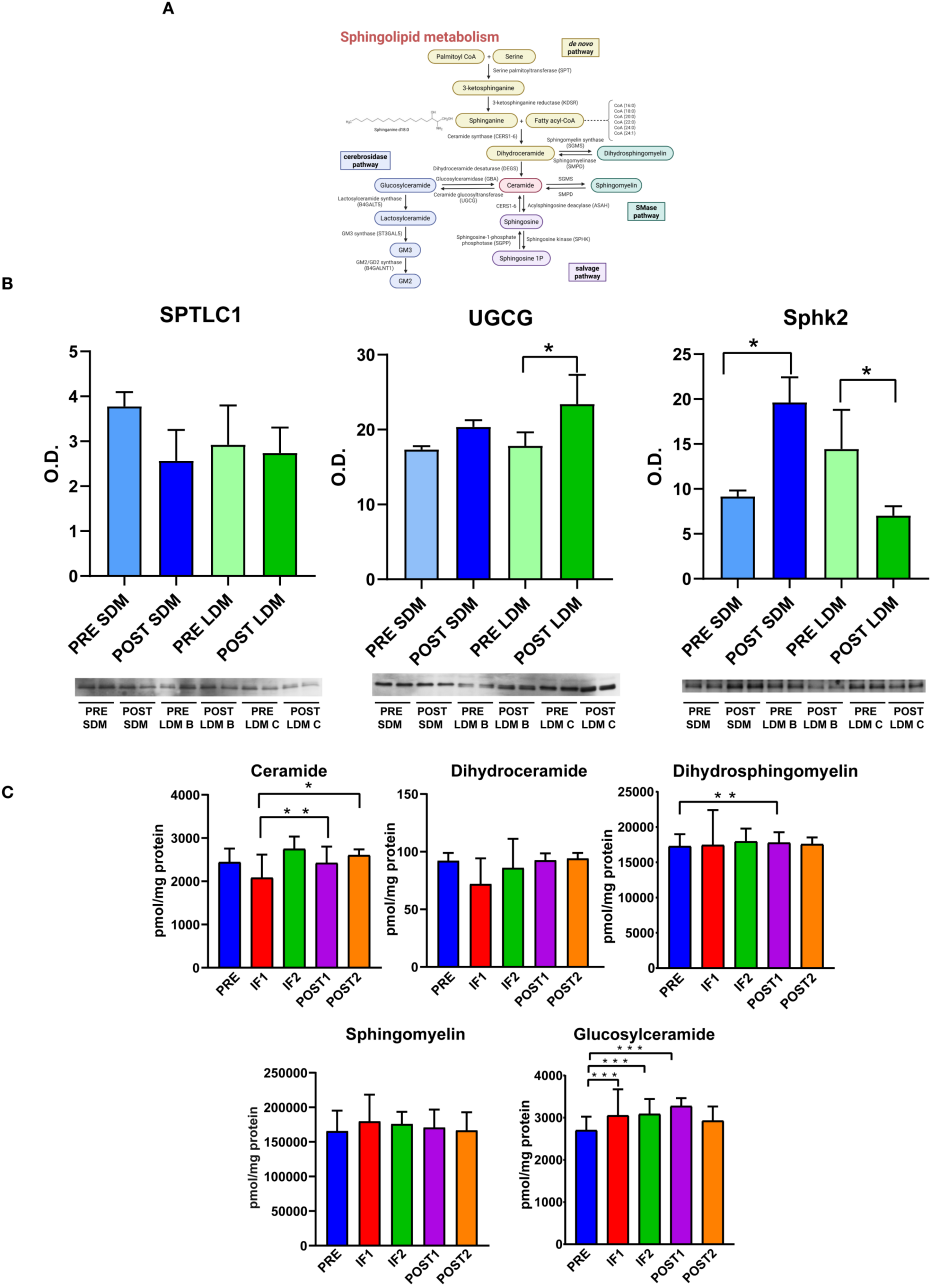
Serum sphingolipid analysis. **(A)** Sphingolipid metabolism pathway. **(B)** Representative bar graphs (means ± SD) and corresponding immunoblot images showing quantitative pre-flight and post-flight levels of SPTLC1, serine palmitoyl transferase 1; glucosylceramide synthase (UGCG), and SPHK2, sphingosine kinase 2 in skeletal muscle of astronauts exposed to short-duration (9 days, SDM) and long-duration (6 months, LDM) missions. Data were normalized to the total protein load visualized with Sypro Ruby. O.D. = optical density; * indicates a significant difference (*p < 0.05, **p < 0.01, ***p < 0.001). Statistical analysis was performed using a paired t-test, with significance set at p < 0.05. Full-length blot images are provided in **Supplementary Figure S1**. **(C)** Quantification of serum sphingolipid levels in pre-flight (PRE), in-flight (IF1, IF2), and post-flight (POST1, POST2) samples using targeted MRM analysis.

Results are presented in **Figure 5**. Panel A illustrates the sphingolipid metabolism pathway, while Panel B shows immunoblotting results for selected enzymes involved in sphingolipid metabolism from muscle extracts. Due to the limited availability of muscle extracts, three key enzymes were selected from the entire pathway:

- SPTLC1, a fundamental enzyme catalyzing the rate-limiting first step of *de novo* sphingolipid synthesis.
- UCGC, a key enzyme responsible for initiating glycosphingolipid (GSL) biosynthesis.
- SPHK2, that phosphorylates sphingosine to produce sphingosine-1-phosphate (S1P) and is localized in the nucleus, endoplasmic reticulum, and mitochondria.

In astronauts exposed to prolonged microgravity (6 months, LDM), panel B shows no changes in SPTLC1 levels between PRE- and POST-flight, suggesting that *de novo* sphingolipid synthesis is maintained. UCGC levels increased in POST-flight, suggesting increased glycosphingolipid production, whereas SPHK2 levels decreased. Interestingly, in astronauts exposed to 9 days of microgravity without countermeasures (SDM), SPTLC1 and UGCG levels remained unchanged, while SPHK2 levels increased, suggesting that acute exposure to microgravity in absence of inflight countermeasures may have impacted sphingolipid signaling.

Panel C shows serum levels of ceramide (Cer), dihydroceramide (dhCer), dihydrosphingomyelin (dhSM), sphingomyelin (SM), and glucosylceramide (GlucCer) across PRE, IF1, IF2, POST1, and POST2 time points, as detected by targeted MRM analysis. Total ceramide decreased in IF1 but rebounded in IF2, POST1, and POST2. Total dhCer and SM remained unchanged, dhSM showed a slight increase, while GlucCer levels rose, consistent with the changes observed in enzyme levels from muscle extracts from astronauts recruited in a previous mission (Blottner et al., 2023).

## 4 Discussion

The challenge of monitoring how the human body reacts to the space environment, especially during future planned long-duration missions, is crucial for ensuring the safety and health of astronauts.

The present research aimed to identify specific low-abundant biomarkers that can effectively monitor changes in astronaut’s muscle and bone physiology resulting from prolonged exposure to microgravity. To capture a comprehensive picture of these physiological adaptations, we analyzed serum samples (collected before and after a six-month duration flight) utilizing a robust automatic set up based on nanoparticles able to capture also low abundant proteins.

Specifically, the Proteograph™ methodology enables a more detailed profiling of serum proteins compared to similar technologies (Beimers et al., 2025; Roger et al., 2025). Our study builds on previous human spaceflight research that has primarily relied on multiomics analyses (Garrett-Bakelman et al., 2019) or traditional prefractionation proteomic approaches (Kimura et al., 2024), thereby broadening current knowledge of how microgravity influences protein expression associated with human adaptation mechanisms in space.

Our findings revealed that 11 proteins, listed in **Table 1**, exhibited significant changes as a result of spaceflight (POST1 *vs*. PRE). Notably, three of these proteins were further analyzed by an independent technology in all time points, reinforcing the reliability of our initial proteomic data. The data obtained from astronaut serum indicated substantial remodeling of ECM components and alterations in lipid metabolism, consistent with trends previously observed in proteomic analyses of astronaut skeletal muscle tissue (Blottner et al., 2023). Furthermore, we used IPA to explore the pathways associated with the proteins that changed post-flight. This analysis identified ten dysregulated pathways, related to muscle function, including those related to lipid metabolism, neuroplasticity and neuromuscular signaling, and semaphorin signaling. Interestingly, we did not observe any evidence of ER stress. Our proteomic analysis reveals also one pathway related to brain function. Increased levels of RELN and decreased levels of BDNF, hypoxanthine phosphoribosyltransferase 1 (HPRT1), TNF Superfamily Member 12 and 13 (TNFSF12 and TNFSF13) indicated that protective mechanisms are activated apparently assisting in brain function and counteracting neuronal apoptosis. Previous studies on BDNF plasma levels in the bed-rest spaceflight analogue (Passaro et al., 2017) and proteomic profiling of the secretome from space travellers during the SpaceX Inspiration4 mission (Houerbi et al., 2024) showed, already after only three days in space, evidence consistent with early in-flight neuroplasticity biomarkers such as RELN and BDNF, findings that align with the biomarker patterns observed in our serum samples. Collectively, these studies support a strong interrelationship between BDNF serum levels, neural integrity, and skeletal muscle health in astronauts, underscoring the need to optimize in-flight countermeasures against cognitive decline and neuroplasticity impairment (Marfia et al., 2022). In serum, the pathway associated with lipoprotein assembly, metabolism, and clearance was activated, accompanied by increased levels of PLIN1, PLIN3, PLIN4. These findings suggest that, in our astronauts, increased levels of circulating perlipins were associated with muscle mass loss and inflammation (Conte et al., 2021).

The increased levels of HDLBP observed during the flight and maintained at both post-flight time points suggest a potential protective role of this protein. HDLBP is involved in several critical cellular functions, including heterochromatin formation, chromosome segregation, and mRNA stability. Decreased levels of this protein have been correlated with impaired DNA damage repair mechanisms, notably in conditions associated with autism (Banday et al., 2021). Importantly, HDLBP functions as an RNA-binding protein that plays a key role in ER translation. It acts as a regulatory factor in gene expression, maintains genome stability, facilitates DNA damage recovery following irradiation, supports mRNA transport and metabolism, and contributes to immune system development, thereby mitigating endothelial stress (Mushtaq et al., 2023). The observed increase in HDLBP levels found in our samples may therefore indicate a protective mechanism aimed at counteracting ER stress, promoting DNA damage repair, and regulating lipid metabolism under spaceflight conditions. At this point elevated HDLBP levels is a novel and significant serum marker that likely contributes to understanding how astronauts counteract challenges associated with ER stress, DNA damage, and lipid metabolism disruptions linked to musculoskeletal adaptation in spaceflight.

To get further insight into the presence of circulating biomarkers able to monitor muscle adaptation during long duration spaceflight, we took advantage from our and other previous proteomic studies on muscle extracts that indicated a fingerprint at tissue level. We found a correlation with signals from blood including sphingolipid signalling (Rittweger et al., 2018; Blottner et al., 2023) (Murgia et al., 2024). In our recent proteomic analysis of pre- and post-flight muscle tissues, we noted that the inhibition of several metabolic pathways, including the TCA cycle and mitochondrial fatty acid oxidation, suggest dysfunctions in muscle cell lipid synthesis and degradation. Such impairments could lead to the accumulation of intramuscular adipose tissue (IMAT) and intramyocellular lipid droplets (myosteatosis) with reduced muscle quality and insulin resistance following disuse reported from several analogue studies (Manini et al., 2007; Mason et al., 2014; Eggelbusch et al., 2024).

Further exploratory investigation focused on changes of enzymes regulating sphingolipid metabolism in muscle tissue, available from a previous study, after 6 months of spaceflight, thus highlighting the increase in UCGC and decrease of the SPHK2 levels. The latter is known to regulate a variety of cellular processes, including mitochondrial respiration (Diaz Escarcega et al., 2021). Decreased levels of this enzyme in POST muscle extracts from astronauts may be associated with mitochondrial dysfunction, as found in the present study and even more reported by others (Blottner et al., 2023; Murgia et al., 2024). Furthermore, the increased levels of UCGC detected in muscle tissue from astronauts involved in a previous mission (Blottner et al., 2023) support the increase in GlcCer levels observed in astronaut sera during spaceflight. An initial reduction in Cer levels, as observed in our subjects, may be explained with enhanced Cer flux through the GlcCer metabolic pathway (Dodge et al., 2015). This increase of GlcCer levels could be associated with changes in muscle fiber metabolic phenotype, as well as alterations in contractility and fatigability (Henriques et al., 2015). Our findings indicate a disrupted GlcCer homeostasis in astronauts, leading to the generally accepted hypothesis that such alterations likely contribute to the loss of muscle strength non-mitigated in astronauts by physical countermeasures currently available on the ISS.

Despite the compelling results, this study has some limitations which belong to the well-known constraints to human space experiments: 1) only small volumes of serum samples were available; 2) restricted amount of participants from astronaut corps included in the study; 3) limited number of blood draws inflight (two time points); 4) individual exercise data other than those previously reported as general overview (e.g., number of days of exercise/devices used; see Schoenrock et al., 2024) were unavailable for this study. However currently available physical countermeasure protocols (see 2.3 Methods) are routinely followed by all astronauts onboard the International Space Station (Scott et al., 2023) suggesting nearly comparable exercise conditions of the astronaut blood donors included to this study.

As we move toward increasingly ambitious missions, it is essential to design effective countermeasures against the deleterious effects of the space exposome on human physiology adaptation (Patel et al., 2020) to design more personalized sensimotor and neuromuscular-driven countermeasure protocols (Schmidt and Goodwin, 2013; Macaulay et al., 2021; Bailey et al., 2025).

Identifying novel serum biomarkers from apparently inconspicuous small changes in low-abundant serum protein levels found in small blood samples easily obtainable from astronauts even under operational constraints is an encouraging new step forward to further understanding complexity of biological pathways from systemic, organ-based to tissue and cells, more or less affected mutually by spaceflight. Precision analyses used to define yet underexplored serum biomarkers from astronaut blood in more detail will likely enhance our capability (i) to improve space-related biomedical monitoring, (ii) to better design interventions able to enhance mission-related astronaut health and performance control and (iii) eventually finding efficient mitigation strategies in future health risk assessment. Alternative technologies in biomedical monitoring such as onboard health monitoring and miniaturization of biosensor technology (Reinsch et al., 2022) will become even more important particularly with a more integrative focus on countermeasure protocols available for applications beyond low Earth orbit (Bailey et al., 2025). Overall, this research offers valuable insights from the emerging field of space medicine that could benefit not only for astronauts in future missions but also for wider applications in the field of human physiology and extreme environments on Earth.

## Data Availability

The datasets presented in this study can be found in online repositories. The names of the repository/repositories and accession number(s) can be found below: https://www.ebi.ac.uk/pride/archive/, PXD069732.

## Glossary

ACTN4: Actinin alpha 4
ALPL: Alkaline phosphatase
APOA: Apolipoprotein A1
APOB: Apoliprotein B
BDNF: Brain-derived neurotrophic factor
Cer: Ceramide
COL11A2: Collagen alpha-2(XI) chain
COL1A1: Collagen alpha-1(I) chain
COL3A1: Collagen alpha-1(III) chain
COL6A1: Collagen type VI alpha 1 chain
COL6A2: Collagen type VI alpha 2 chain
COL6A3: Collagen type VI alpha 3 chain
CTHRC1: collagen triple helix repeat containing 1
dhCer: Dihydroceramide
dhSM: Dihydrosphingomyelin
DIA: Data Independent Acquisition
ECM: Extracellular matrix
ER: Endoplasmic reticulum
ESA: European Space Agency
FDR: False discovery rates
GlucCer: Glucosylceramide
HDL: High-density lipoproteins
HDLBP: HDL binding protein
HPR: Haptoglobin-related protein
IF1: in-flight 1 flight days 31-60
IF2: in-flight 210 days before return to Earth
IPA: Ingenuity Pathway Analysis
ISS: International Space Station
LC-MS/MS: Liquid Chromatography coupled with Mass Spectrometry
LDM: Long duration mission
MYL1: Myosin light chain 1
MYL11: Myosin regulatory light chain 11
MRM-MS: Multiple-Reaction Monitoring Mass Spectrometry
NASA: National Aeronautics and Space Administration
NPs: Nanoparticles
OMD: Osteomodulin
OPN: Osteopontin
PCSK1: Neuroendocrine convertase 1
PF4: Platelet factor 4
PLIN1: Perilipin-1
PLIN3: Perilipin-3
PLIN4: Perilipin-4
POST1: post-flight 13–5 days after landing
POST2: post-flight 2 105 days after landing
POSTN: Periostin
PRE: pre flight
RELN: Reelin
S1P: Sphingosine-1-phosphate
SDM: Short duration mission
SM: Sphingomyelin
SPHK2: Sphingosine kinase 2
SPON2: Spondin-2
SPTLC1: Serine palmitoyltransferase 1
TNNI1: Troponin I
UGCG: Glucosylceramide synthase
VIM: Vimentin.

## References

[B1] Al-Daghri N. M. Torretta E. Barbacini P. Asare H. Ricci C. Capitanio D. . (2020). Publisher correction: Sphingolipid serum profiling in vitamin D deficient and dyslipidemic obese dimorphic adults. Sci. Rep. 10, 2183. doi: 10.1038/s41598-020-58287-x. PMID: 32019944 PMC7000704

[B2] Anderson N. L. Anderson N. G. (2002). The human plasma proteome. Mol. Cell. Proteomics 1, 845–867. doi: 10.1074/mcp.R200007-MCP200. PMID: 12488461

[B3] Bailey D. M. Blottner D. Gunga H.-C. Schneider S. Wotring V. Baatout S. . (2025). Integrative focus on the space exposome-integrome: physiological challenges and practical limits of countermeasures beyond low Earth orbit. NPJ Microgravity 11, 82. doi: 10.1038/s41526-025-00537-1. PMID: 41266377 PMC12635128

[B4] Banday S. Pandita R. K. Mushtaq A. Bacolla A. Mir U. S. Singh D. K. . (2021). Autism-associated vigilin depletion impairs DNA damage repair. Mol. Cell. Biol. 41, e0008221. doi: 10.1128/MCB.00082-21. PMID: 33941620 PMC8224237

[B5] Barbacini P. Blottner D. Capitanio D. Trautmann G. Block K. Torretta E. . (2022a). Effects of omega-3 and antioxidant cocktail supplement on prolonged bed rest: Results from serum proteome and sphingolipids analysis. Cells 11, 2120. doi: 10.3390/cells11132120. PMID: 35805205 PMC9266137

[B6] Barbacini P. Casas J. Torretta E. Capitanio D. Maccallini G. Hirschler V. . (2019). Regulation of serum sphingolipids in Andean children born and living at high altitude, (3775 m). Int. J. Mol. Sci. 20, 2835. doi: 10.3390/ijms20112835. PMID: 31212599 PMC6600227

[B7] Barbacini P. Torretta E. Arosio B. Ferri E. Capitanio D. Moriggi M. . (2022b). Novel insight into the serum sphingolipid fingerprint characterizing longevity. Int. J. Mol. Sci. 23, 2428. doi: 10.3390/ijms23052428. PMID: 35269570 PMC8910653

[B8] Beimers W. F. Overmyer K. A. Sinitcyn P. Lancaster N. M. Quarmby S. T. Coon J. J. (2025). Technical evaluation of plasma proteomics technologies. J. Proteome Res. 24, 3074–3087. doi: 10.1021/acs.jproteome.5c00221. PMID: 40366296 PMC12150332

[B9] Binder H. Wirth H. Arakelyan A. Lembcke K. Tiys E. S. Ivanisenko V. A. . (2014). Time-course human urine proteomics in space-flight simulation experiments. BMC Genomics 15, S2. doi: 10.1186/1471-2164-15-S12-S2. PMID: 25563515 PMC4303941

[B10] Blottner D. Moriggi M. Trautmann G. Furlan S. Block K. Gutsmann M. . (2024). Nitrosative stress in astronaut skeletal muscle in spaceflight. Antioxidants 13, 432. doi: 10.3390/antiox13040432. PMID: 38671880 PMC11047620

[B11] Blottner D. Moriggi M. Trautmann G. Hastermann M. Capitanio D. Torretta E. . (2023). Space omics and tissue response in astronaut skeletal muscle after short and long duration missions. Int. J. Mol. Sci. 24, 4095. doi: 10.3390/ijms24044095. PMID: 36835504 PMC9962627

[B12] Blume J. E. Manning W. C. Troiano G. Hornburg D. Figa M. Hesterberg L. . (2020). Rapid, deep and precise profiling of the plasma proteome with multi-nanoparticle protein corona. Nat. Commun. 11, 3662. doi: 10.1038/s41467-020-17033-7. PMID: 32699280 PMC7376165

[B13] Brzhozovskiy A. Kononikhin A. Indeykina M. Pastushkova L. Popov I. Nikolaev E. . (2017). Label-free study of cosmonaut’s urinary proteome changes after long-duration spaceflights. Eur. J. Mass Spectrom. 23, 225–229. doi: 10.1177/1469066717717610. PMID: 29028400

[B14] Çelen İ. Jayasinghe A. Doh J. H. Sabanayagam C. R. (2023). Transcriptomic signature of the simulated microgravity response in Caenorhabditis elegans and comparison to spaceflight experiments. Cells 12, 270. doi: 10.3390/cells12020270. PMID: 36672205 PMC9856674

[B15] Chandrasekaran P. Weiskirchen S. Weiskirchen R. (2024). Perilipins: A family of five fat‐droplet storing proteins that play a significant role in fat homeostasis. J. Cell. Biochem. 125, e30579. doi: 10.1002/jcb.30579. PMID: 38747370

[B16] Conte M. Santoro A. Collura S. Martucci M. Battista G. Bazzocchi A. . (2021). Circulating perilipin 2 levels are associated with fat mass, inflammatory and metabolic markers and are higher in women than men. Aging 13, 7931–7942. doi: 10.18632/aging.202840. PMID: 33735111 PMC8034884

[B17] Demichev V. Messner C. B. Vernardis S. I. Lilley K. S. Ralser M. (2020). DIA-NN: neural networks and interference correction enable deep proteome coverage in high throughput. Nat. Methods 17, 41–44. doi: 10.1038/s41592-019-0638-x. PMID: 31768060 PMC6949130

[B18] Diaz Escarcega R. McCullough L. D. Tsvetkov A. S. (2021). The functional role of sphingosine kinase 2. Front. Mol. Biosci. 8. doi: 10.3389/fmolb.2021.683767. PMID: 34055895 PMC8160245

[B19] Dodge J. C. Treleaven C. M. Pacheco J. Cooper S. Bao C. Abraham M. . (2015). Glycosphingolipids are modulators of disease pathogenesis in amyotrophic lateral sclerosis. Proc. Natl. Acad. Sci. 112, 8100–8105. doi: 10.1073/pnas.1508767112. PMID: 26056266 PMC4491749

[B20] Eggelbusch M. Charlton B. T. Bosutti A. Ganse B. Giakoumaki I. Grootemaat A. E. . (2024). The impact of bed rest on human skeletal muscle metabolism. Cell Rep. Med. 5, 101372. doi: 10.1016/j.xcrm.2023.101372. PMID: 38232697 PMC10829795

[B21] Fava M. De Dominicis N. Forte G. Bari M. Leuti A. Maccarrone M. (2024). Cellular and molecular effects of microgravity on the immune system: A focus on bioactive lipids. Biomolecules 14, 446. doi: 10.3390/biom14040446. PMID: 38672462 PMC11048039

[B22] Gabel L. Liphardt A.-M. Hulme P. A. Heer M. Zwart S. R. Sibonga J. D. . (2022). Incomplete recovery of bone strength and trabecular microarchitecture at the distal tibia 1 year after return from long duration spaceflight. Sci. Rep. 12, 9446. doi: 10.1038/s41598-022-13461-1. PMID: 35773442 PMC9247070

[B23] Garrett-Bakelman F. E. Darshi M. Green S. J. Gur R. C. Lin L. Macias B. R. . (2019). The NASA Twins Study: A multidimensional analysis of a year-long human spaceflight. Sci. (1979) 364, eaau8650. doi: 10.1126/science.aau8650. PMID: 30975860 PMC7580864

[B24] Genah S. Monici M. Morbidelli L. (2021). The effect of space travel on bone metabolism: Considerations on today’s major challenges and advances in pharmacology. Int. J. Mol. Sci. 22, 4585. doi: 10.3390/ijms22094585. PMID: 33925533 PMC8123809

[B25] Hatton I. A. Galbraith E. D. Merleau N. S. C. Miettinen T. P. Smith B. M. Shander J. A. (2023). The human cell count and size distribution. Proc. Natl. Acad. Sci. 120, e2303077120. doi: 10.1073/pnas.2303077120. PMID: 37722043 PMC10523466

[B26] Henriques A. Croixmarie V. Priestman D. A. Rosenbohm A. Dirrig-Grosch S. D’Ambra E. . (2015). Amyotrophic lateral sclerosis and denervation alter sphingolipids and up-regulate glucosylceramide synthase. Hum. Mol. Genet. 24, 7390–7405. doi: 10.1093/hmg/ddv439. PMID: 26483191 PMC4664174

[B27] Houerbi N. Kim J. Overbey E. G. Batra R. Schweickart A. Patras L. . (2024). Secretome profiling reveals acute changes in oxidative stress, brain homeostasis, and coagulation following short-duration spaceflight. Nat. Commun. 15, 4862. doi: 10.1038/s41467-024-48841-w. PMID: 38862464 PMC11166969

[B28] Huang T. Wang J. Stukalov A. Donovan M. K. R. Ferdosi S. Williamson L. . (2023). Protein coronas on functionalized nanoparticles enable quantitative and precise large-scale deep plasma proteomics. Biorxiv, Cold Spring Harbor Laboratory Press, NY. doi: 10.1101/2023.08.28.555225, PMID:

[B29] Ji Y. Zhang Q. Li H. Chen L. Wu Y. Lin S. (2025). Platelet factor 4: A mysterious chemokine in inflammatory regulation diseases. J. Inflamm. Res. 18, 4481–4495. doi: 10.2147/JIR.S504673. PMID: 40166592 PMC11956735

[B30] Keshishian H. Burgess M. W. Specht H. Wallace L. Clauser K. R. Gillette M. A. . (2017). Quantitative, multiplexed workflow for deep analysis of human blood plasma and biomarker discovery by mass spectrometry. Nat. Protoc. 12, 1683–1701. doi: 10.1038/nprot.2017.054. PMID: 28749931 PMC6057147

[B31] Kimura Y. Nakai Y. Ino Y. Akiyama T. Moriyama K. Aiba T. . (2024). Changes in the astronaut serum proteome during prolonged spaceflight. Proteomics 24, e2300328. doi: 10.1002/pmic.202300328. PMID: 38185763

[B32] Knight M. N. Karuppaiah K. Lowe M. Mohanty S. Zondervan R. L. Bell S. . (2018). R-spondin-2 is a Wnt agonist that regulates osteoblast activity and bone mass. Bone Res. 6, 24. doi: 10.1038/s41413-018-0026-7. PMID: 30131881 PMC6089978

[B33] Lin W. Zhu X. Gao L. Mao M. Gao D. Huang Z. (2021). Osteomodulin positively regulates osteogenesis through interaction with BMP2. Cell Death Dis. 12, 147. doi: 10.1038/s41419-021-03404-5. PMID: 33542209 PMC7862363

[B34] Loehr J. A. Guilliams M. E. Petersen N. Hirsch N. Kawashima S. Ohshima H. (2015). Physical training for long-duration spaceflight. Aerosp. Med. Hum. Perform. 86, A14–A23. doi: 10.3357/AMHP.EC03.2015. PMID: 26630191

[B35] Macaulay T. R. Peters B. T. Wood S. J. Clément G. R. Oddsson L. Bloomberg J. J. (2021). Developing proprioceptive countermeasures to mitigate postural and locomotor control deficits after long-duration spaceflight. Front. Syst. Neurosci. 15. doi: 10.3389/fnsys.2021.658985. PMID: 33986648 PMC8111171

[B36] Manini T. M. Clark B. C. Nalls M. A. Goodpaster B. H. Ploutz-Snyder L. L. Harris T. B. (2007). Reduced physical activity increases intermuscular adipose tissue in healthy young adults. Am. J. Clin. Nutr. 85, 377–384. doi: 10.1093/ajcn/85.2.377. PMID: 17284732

[B37] Manis C. Murgia A. Manca A. Pantaleo A. Cao G. Caboni P. (2023). Untargeted lipidomics of erythrocytes under simulated microgravity conditions. Int. J. Mol. Sci. 24, 4379. doi: 10.3390/ijms24054379. PMID: 36901810 PMC10002504

[B38] Marfia G. Navone S. E. Guarnaccia L. Campanella R. Locatelli M. Miozzo M. . (2022). Space flight and central nervous system: Friends or enemies? Challenges and opportunities for neuroscience and neuro‐oncology. J. Neurosci. Res. 100, 1649–1663. doi: 10.1002/jnr.25066. PMID: 35678198 PMC9544848

[B39] Mason R. R. Meex R. C. R. Russell A. P. Canny B. J. Watt M. J. (2014). Cellular localization and associations of the major lipolytic proteins in human skeletal muscle at rest and during exercise. PloS One 9, e103062. doi: 10.1371/journal.pone.0103062. PMID: 25054327 PMC4108417

[B40] Monti E. Reggiani C. Franchi M. V. Toniolo L. Sandri M. Armani A. . (2021). Neuromuscular junction instability and altered intracellular calcium handling as early determinants of force loss during unloading in humans. J. Physiol. 599, 3037–3061. doi: 10.1113/JP281365. PMID: 33881176 PMC8359852

[B41] Murgia M. Ciciliot S. Nagaraj N. Reggiani C. Schiaffino S. Franchi M. V. . (2022). Signatures of muscle disuse in spaceflight and bed rest revealed by single muscle fiber proteomics. PNAS Nexus 1, pgac086. doi: 10.1093/pnasnexus/pgac086. PMID: 36741463 PMC9896895

[B42] Murgia M. Rittweger J. Reggiani C. Bottinelli R. Mann M. Schiaffino S. . (2024). Spaceflight on the ISS changed the skeletal muscle proteome of two astronauts. NPJ Microgravity 10, 60. doi: 10.1038/s41526-024-00406-3. PMID: 38839773 PMC11153545

[B43] Mushtaq A. Mir U. S. Altaf M. (2023). Multifaceted functions of RNA-binding protein vigilin in gene silencing, genome stability, and autism-related disorders. J. Biol. Chem. 299, 102988. doi: 10.1016/j.jbc.2023.102988. PMID: 36758804 PMC10011833

[B44] Nanjappa V. Thomas J. K. Marimuthu A. Muthusamy B. Radhakrishnan A. Sharma R. . (2014). Plasma proteome database as a resource for proteomics research: 2014 update. Nucleic Acids Res. 42, D959–D965. doi: 10.1093/nar/gkt1251. PMID: 24304897 PMC3965042

[B45] Nielsen M. J. Petersen S. V. Jacobsen C. Oxvig C. Rees D. Møller H. J. . (2006). Haptoglobin-related protein is a high-affinity hemoglobin-binding plasma protein. Blood 108, 2846–2849. doi: 10.1182/blood-2006-05-022327. PMID: 16778136

[B46] Passaro A. Soavi C. Marusic U. Rejc E. Sanz J. M. Morieri M. L. . (2017). Computerized cognitive training and brain derived neurotrophic factor during bed rest: mechanisms to protect individual during acute stress. Aging 9, 393–407. doi: 10.18632/aging.101166. PMID: 28161695 PMC5361671

[B47] Patel Z. S. Brunstetter T. J. Tarver W. J. Whitmire A. M. Zwart S. R. Smith S. M. . (2020). Red risks for a journey to the red planet: The highest priority human health risks for a mission to Mars. NPJ Microgravity 6, 33. doi: 10.1038/s41526-020-00124-6. PMID: 33298950 PMC7645687

[B48] Ramos-Molina B. Martin M. G. Lindberg I. (2016). PCSK1 variants and human obesity. Prog. Mol. Biol. Transl. Sci. 140, 47–74. doi: 10.1016/bs.pmbts.2015.12.001, PMID: 27288825 PMC6082390

[B49] Reinsch N. Füting A. Höwel D. Neven K. (2022). The BIOMONITOR III Injectable Cardiac Monitor: Clinical experience with a novel injectable cardiac monitor. J. Clin. Med. 11, 1634. doi: 10.3390/jcm11061634. PMID: 35329960 PMC8954265

[B50] Rittweger J. Albracht K. Flück M. Ruoss S. Brocca L. Longa E. . (2018). Sarcolab pilot study into skeletal muscle’s adaptation to long-term spaceflight. NPJ Microgravity 4, 18. doi: 10.1038/s41526-018-0052-1. PMID: 30246141 PMC6141586

[B51] Roger K. Metatla I. Ceccacci S. Wahbi K. Motté L. Chhuon C. . (2025). Mining the plasma proteome: Evaluation of enrichment methods for depth and reproducibility. J. Proteomics 321, 105519. doi: 10.1016/j.jprot.2025.105519. PMID: 40829696

[B52] Schaefer E. J. Anthanont P. Asztalos B. F. (2014). High-density lipoprotein metabolism, composition, function, and deficiency. Curr. Opin. Lipidol. 25, 194–199. doi: 10.1097/MOL.0000000000000074. PMID: 24785961 PMC5489068

[B53] Schmidt M. A. Goodwin T. J. (2013). Personalized medicine in human space flight: Using omics based analyses to develop individualized countermeasures that enhance astronaut safety and performance. Metabolomics 9, 1134–1156. doi: 10.1007/s11306-013-0556-3. PMID: 24273472 PMC3825629

[B54] Schoenrock B. Muckelt P. E. Hastermann M. Albracht K. MacGregor R. Martin D. . (2024). Muscle stiffness indicating mission crew health in space. Sci. Rep. 14, 4196. doi: 10.1038/s41598-024-54759-6. PMID: 38378866 PMC10879143

[B55] Scott J. M. Feiveson A. H. English K. L. Spector E. R. Sibonga J. D. Dillon E. L. . (2023). Effects of exercise countermeasures on multisystem function in long duration spaceflight astronauts. NPJ Microgravity 9, 11. doi: 10.1038/s41526-023-00256-5. PMID: 36737441 PMC9898566

[B56] Scott J. P. R. Weber T. Green D. A. (2020). Editorial: Optimization of exercise countermeasures for human space flight—lessons from terrestrial physiology and operational implementation. Front. Physiol. 10. doi: 10.3389/fphys.2019.01567. PMID: 31998142 PMC6965165

[B57] Tolle G. Serreli G. Deiana M. Moi L. Zavattari P. Pantaleo A. . (2024). Lipidomics of Caco-2 cells under simulated microgravity conditions. Int. J. Mol. Sci. 25, 12638. doi: 10.3390/ijms252312638. PMID: 39684348 PMC11641246

[B58] Torretta E. Arosio B. Barbacini P. Capitanio D. Rossi P. D. Moriggi M. . (2021a). Novel insight in idiopathic normal pressure hydrocephalus (iNPH) biomarker discovery in CSF. Int. J. Mol. Sci. 22, 8034. doi: 10.3390/ijms22158034. PMID: 34360799 PMC8347603

[B59] Torretta E. Arosio B. Barbacini P. Casati M. Capitanio D. Mancuso R. . (2018). Particular CSF sphingolipid patterns identify iNPH and AD patients. Sci. Rep. 8, 13639. doi: 10.1038/s41598-018-31756-0. PMID: 30206302 PMC6133966

[B60] Torretta E. Barbacini P. Al-Daghri N. M. Gelfi C. (2019). Sphingolipids in obesity and correlated co-morbidities: The contribution of gender, age and environment. Int. J. Mol. Sci. 20, 5901. doi: 10.3390/ijms20235901. PMID: 31771303 PMC6929069

[B61] Torretta E. Garziano M. Poliseno M. Capitanio D. Biasin M. Santantonio T. A. . (2021b). Severity of COVID-19 patients predicted by serum sphingolipids signature. Int. J. Mol. Sci. 22, 10198. doi: 10.3390/ijms221910198. PMID: 34638539 PMC8508132

[B62] Tyanova S. Temu T. Sinitcyn P. Carlson A. Hein M. Y. Geiger T. . (2016). The Perseus computational platform for comprehensive analysis of (prote)omics data. Nat. Methods 13, 731–740. doi: 10.1038/nmeth.3901. PMID: 27348712

[B63] Verde L. Galasso M. Coletta D. K. Savastano S. Mandarino L. J. Colao A. . (2025). The interplay of UCP3 and PCSK1 variants in severe obesity. Curr. Obes. Rep. 14, 38. doi: 10.1007/s13679-025-00631-1. PMID: 40281302 PMC12031958

[B64] Zinnall U. Milek M. Minia I. Vieira-Vieira C. H. Müller S. Mastrobuoni G. . (2022). HDLBP binds ER-targeted mRNAs by multivalent interactions to promote protein synthesis of transmembrane and secreted proteins. Nat. Commun. 13, 2727. doi: 10.1038/s41467-022-30322-7. PMID: 35585045 PMC9117268

