# Supplementary material for: Proteograph™-based proteome and sphingolipidome analyses identified novel serum biomarkers to monitor astronauts’ health in spaceflight
Source: Front Physiol. 2026 Apr 22;17:1773221. doi: 10.3389/fphys.2026.1773221 (PMC13143587; doi:10.3389/fphys.2026.1773221)

Supplementary Figure S1. Full-Length Original Blot Images

Serum, 5–14% gradient polyacrylamide gels

Total protein content detected by Sypro Ruby staining

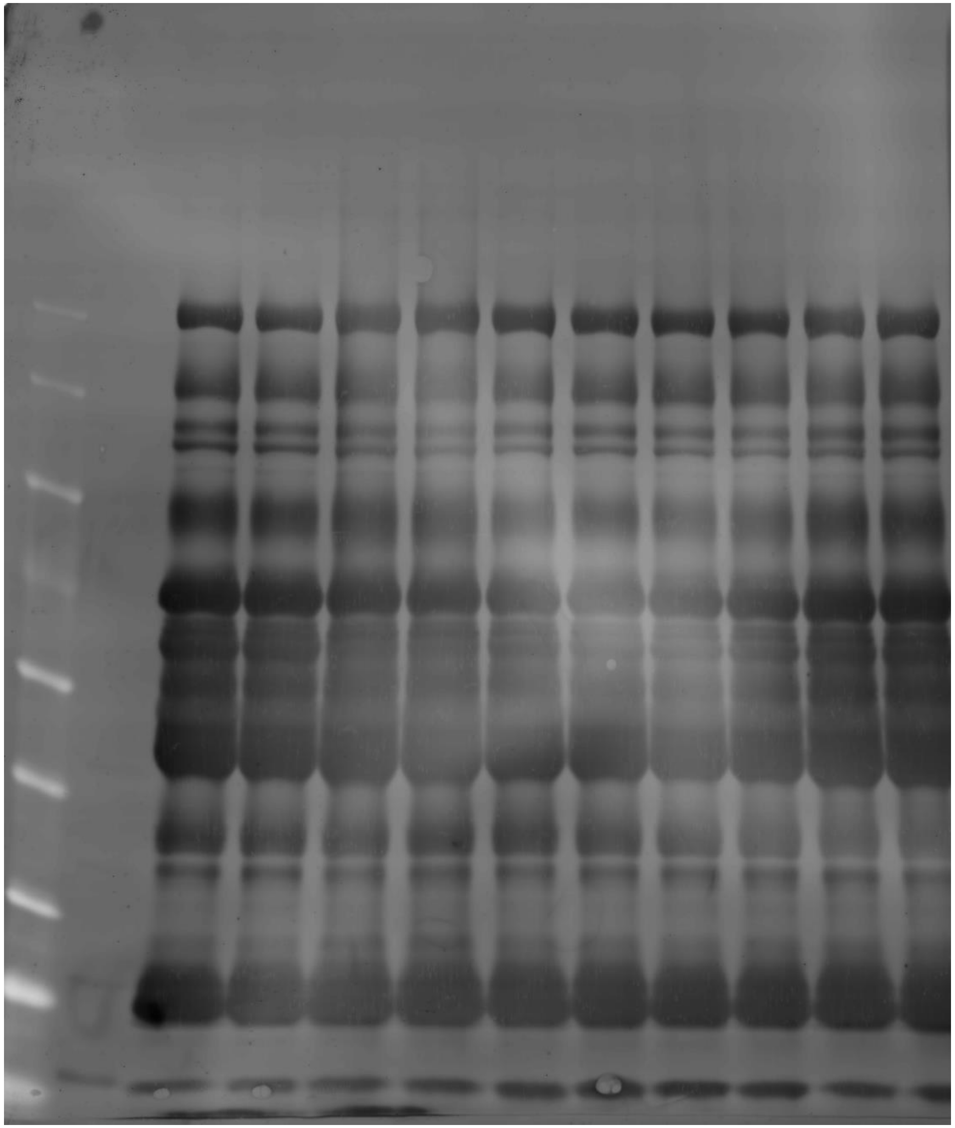

rabbit polyclonal anti-type XI collagen with two alpha chains (COL11A2, 150 kDa), CAB10473 Assay Gene
I 1:1500 BSA 5%
II anti-rabbit 1:10000 TBS-T 1X

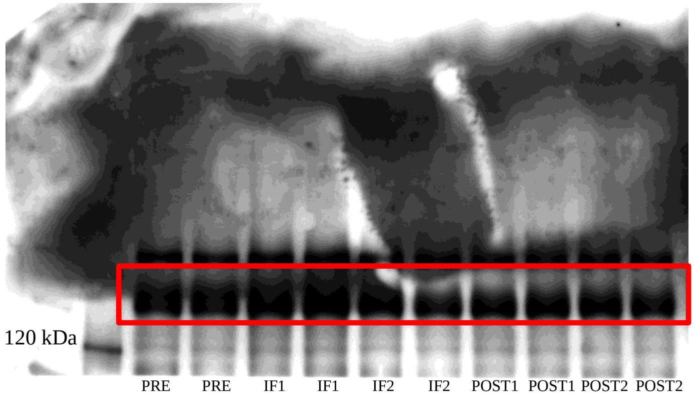

mouse monoclonal anti-collagen triple helix repeat containing 1 (CTHRC1, 28-30 kDa), sc-293270
I 1:500 BSA 5%
II anti-mouse 1:5000 TBS-T 1X

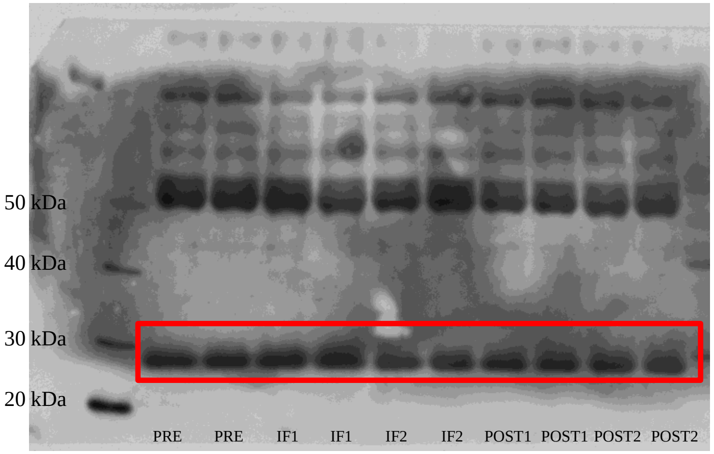

Serum, 5–12% gradient polyacrylamide gels

Total protein content detected by Sypro Ruby staining

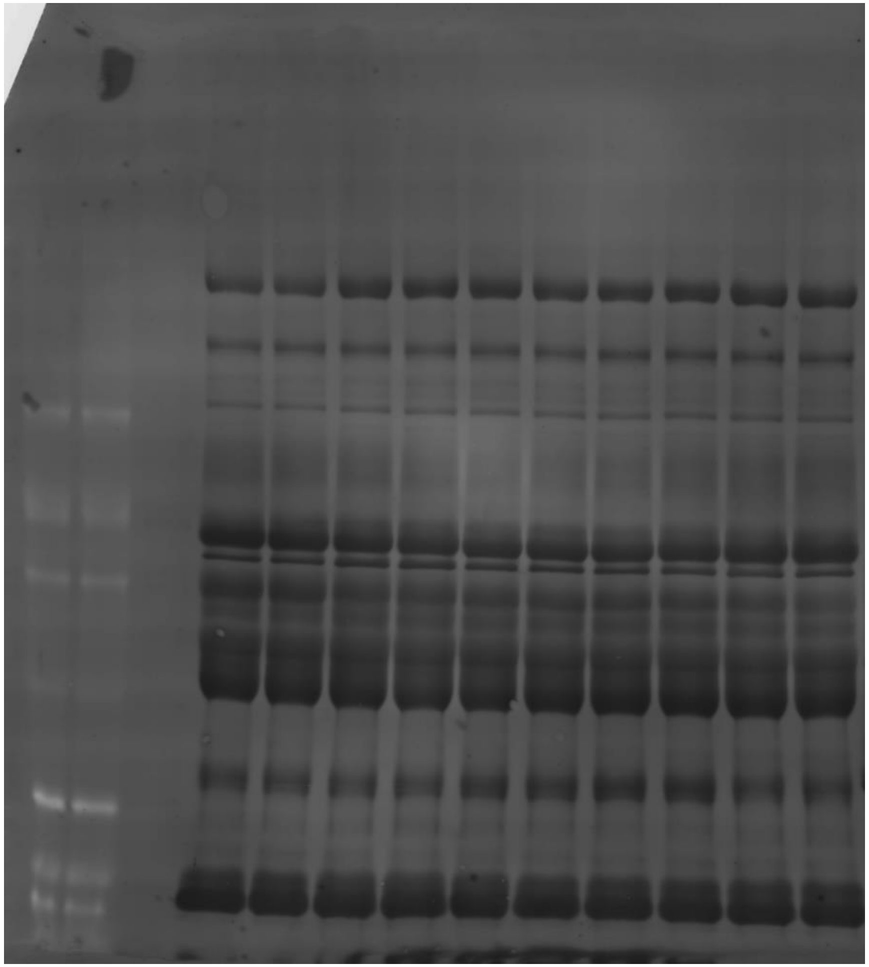

rabbit polyclonal anti-perilipin 4 (PLIN4, 135 kDa) invitrogen
I 1:1000 BSA 5%
II anti-rabbit 1:10000 TBS-T 1X

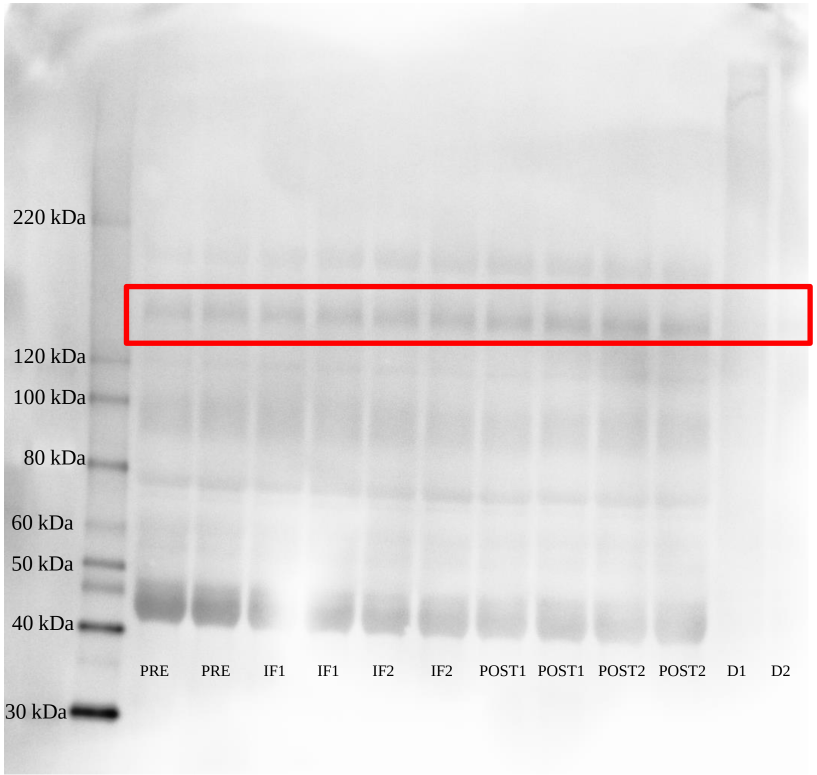

mouse monoclonal anti-HDL binding protein (HDLBP, 155 kDa)
sc-271523
I 1:500 BSA 5%
II anti-mouse 1:5000 TBS-T 1X

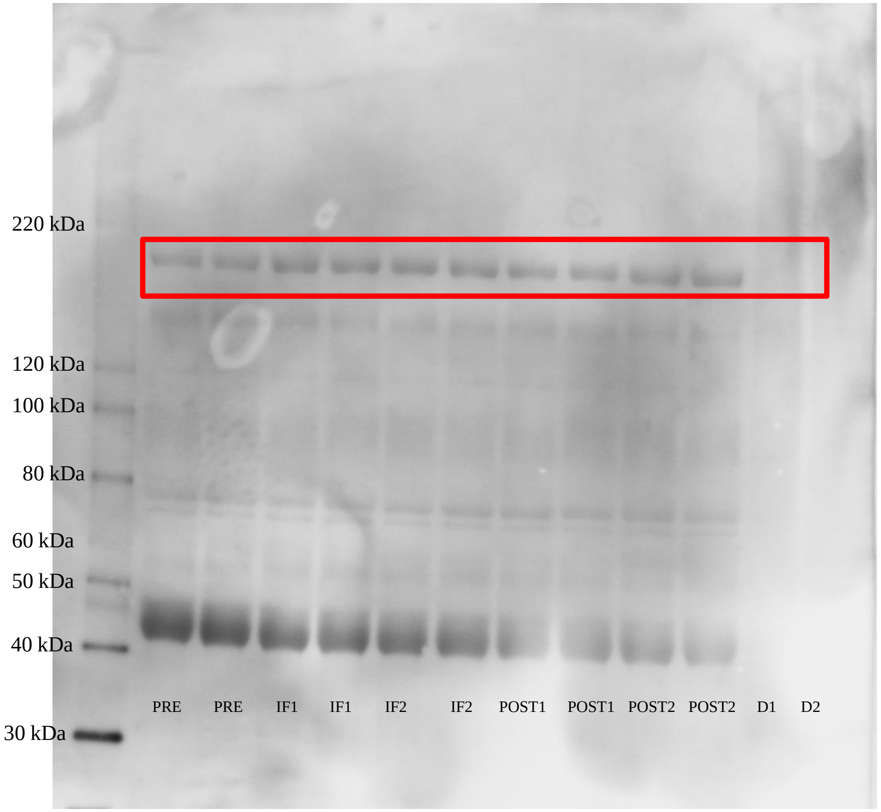

Skeletal muscle, 12–16% gradient polyacrylamide gels

Total protein content detected by Sypro Ruby staining

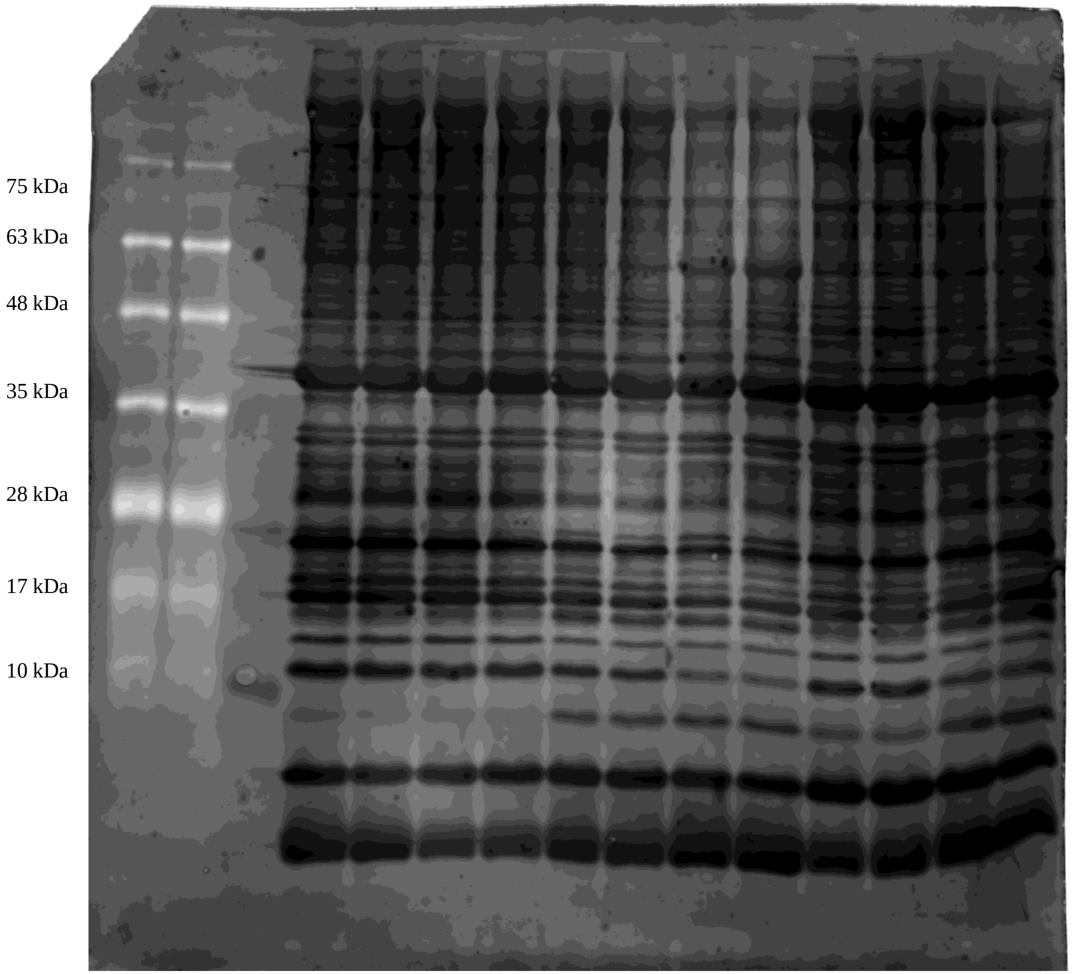

mouse monoclonal anti-serine palmitoyltransferase 1
(SPTLC1, 55 kDa)
sc-374143
I 1:500 BSA 5%
II anti-mouse 1:5000 TBS-T 1X

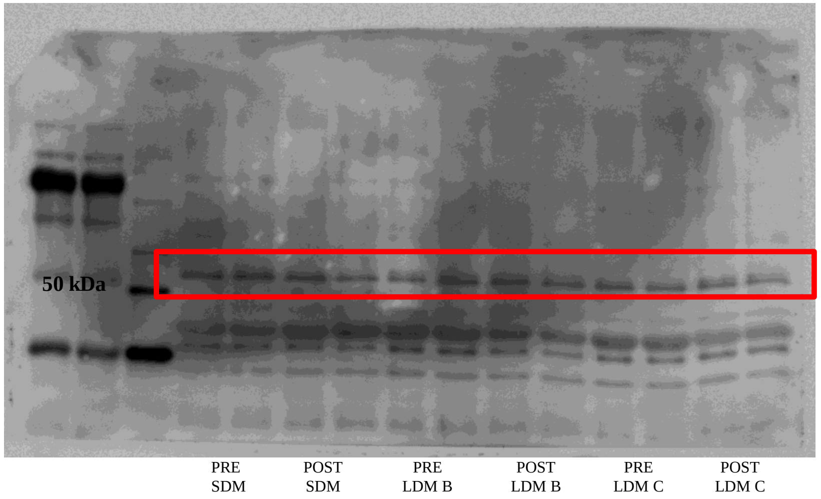

Skeletal muscle, 12–16% gradient polyacrylamide gels

Total protein content detected by Sypro Ruby staining

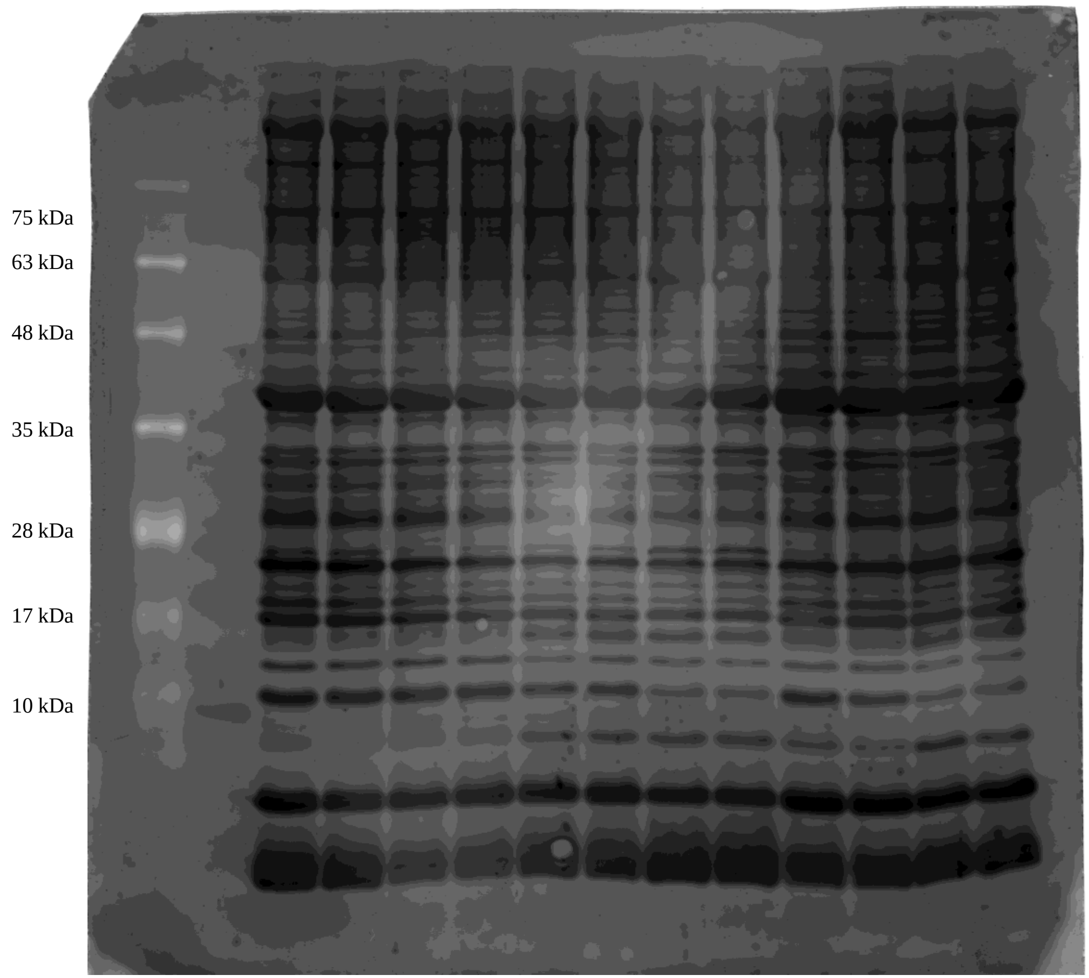

mouse monoclonal anti-sphingosine kinase 2 (Sphk2, 70 kDa) sc-517192
I 1:500 BSA 5%
II anti-mouse 1:5000 TBS-T 1X

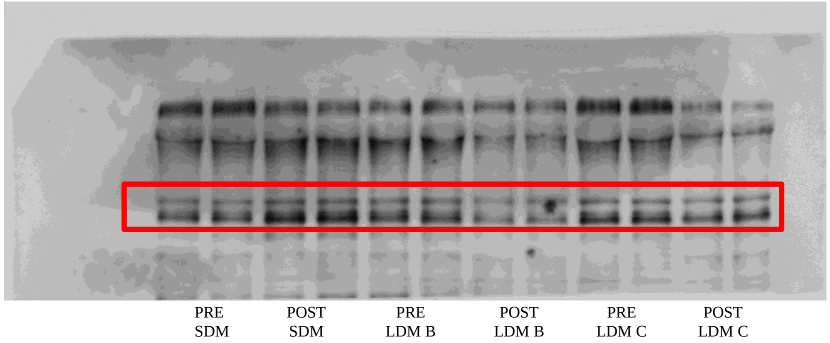

mouse monoclonal anti-glucosylceramide synthase (UGCG, 38 kDa) sc-293235
I 1:500 BSA 5% usato
II anti-mouse 1:5000 TBS-T 1X

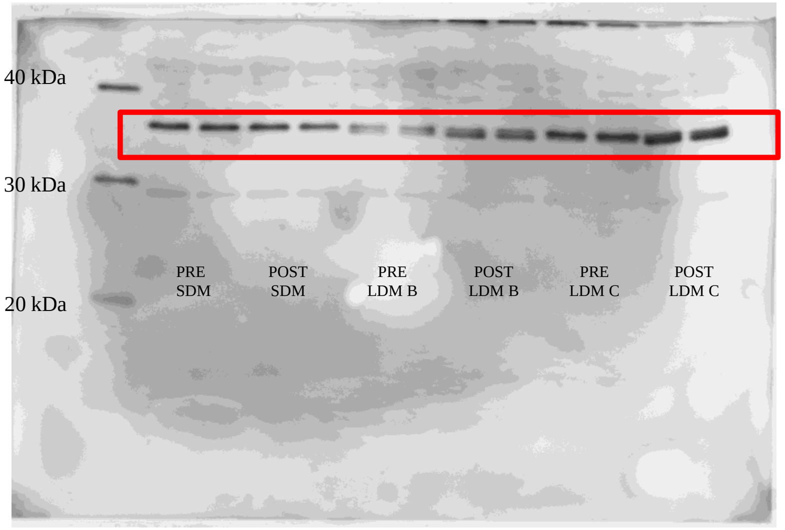

Supplement: Supplementary file 1 [file DataSheet1.pdf]
