# Supplementary material for: Proteograph™-based proteome and sphingolipidome analyses identified novel serum biomarkers to monitor astronauts’ health in spaceflight
Source: Front Physiol. 2026 Apr 22;17:1773221. doi: 10.3389/fphys.2026.1773221 (PMC13143587; doi:10.3389/fphys.2026.1773221)
Supplement: Supplementary file 2 [file DataSheet2.pdf]

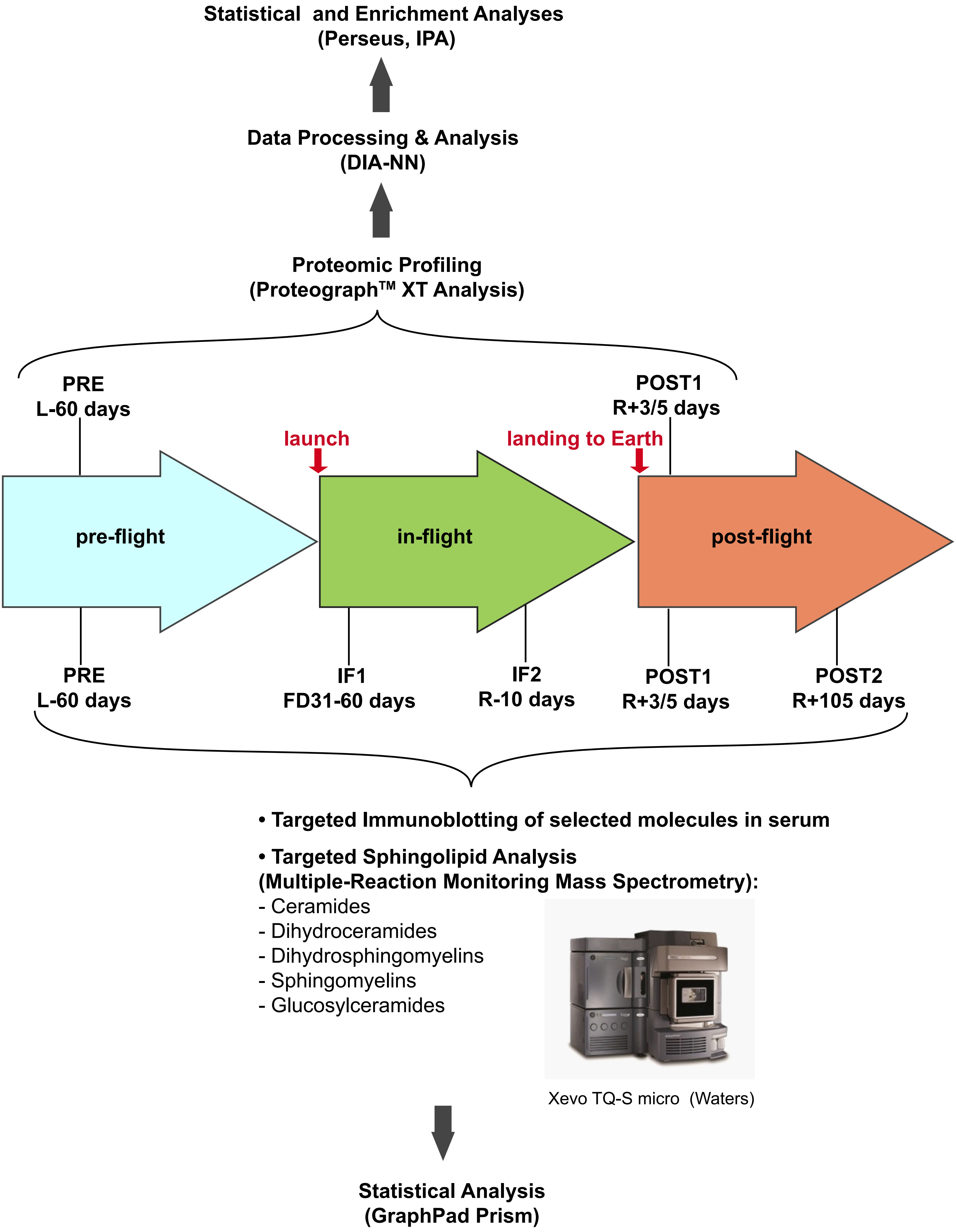

Supplementary Figure S2. Experimental design
