# Supplementary material for: Proteograph™-based proteome and sphingolipidome analyses identified novel serum biomarkers to monitor astronauts’ health in spaceflight
Source: Front Physiol. 2026 Apr 22;17:1773221. doi: 10.3389/fphys.2026.1773221 (PMC13143587; doi:10.3389/fphys.2026.1773221)
Supplement: Supplementary file 3 [file DataSheet3.pdf]

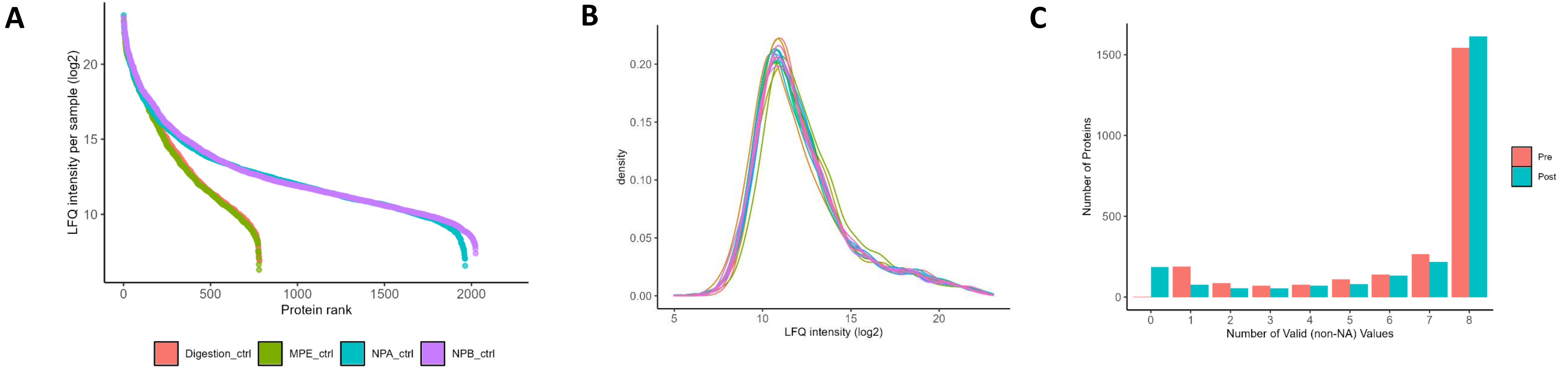

Supplementary Figure S3. Quality control of the proteograph assay analysis.
(A) Ranking plot of protein according to LFQ intensities showing that enrichment was performed successfully.
(B) LFQ intensity distribution per sample that confirmed the good quality of the experiment
(C) Protein count according to their respective number of Valid Values per experimental group
